# DEPDC1B Promotes Melanoma Angiogenesis and Metastasis through Sequestration of Ubiquitin Ligase CDC16 to Stabilize Secreted SCUBE3

**DOI:** 10.1002/advs.202105226

**Published:** 2022-01-27

**Authors:** Feng Hu, Ki On Fong, May Pui Lai Cheung, Jessica Aijia Liu, Rui Liang, Tsz Wai Li, Rakesh Sharma, Philip Pun‐Ching IP, Xintao Yang, Martin Cheung

**Affiliations:** ^1^ School of Biomedical Sciences Li Ka Shing Faculty of Medicine The University of Hong Kong Hong Kong China; ^2^ Department of Neuroscience City University of Hong Kong Tat Chee Avenue Hong Kong China; ^3^ Centre for PanorOmic Sciences Proteomics and Metabolomics Core Facility Li Ka Shing Faculty of Medicine The University of Hong Kong Hong Kong China; ^4^ Department of Pathology Li Ka Shing Faculty of Medicine The University of Hong Kong Hong Kong China

**Keywords:** angiogenesis, CDC16, DEPDC1B, melanoma, SCUBE3

## Abstract

The ability of melanoma to acquire metastasis through the induction of angiogenesis is one of the major causes of skin cancer death. Here, it is found that high transcript levels of *DEP domain containing 1B* (*DEPDC1B*) in cutaneous melanomas are significantly associated with a poor prognosis. Tissue microarray analysis indicates that DEPDC1B expression is positively correlated with SOX10 in the different stages of melanoma. Consistently, DEPDC1B is both required and sufficient for melanoma growth, metastasis, angiogenesis, and functions as a direct downstream target of SOX10 to partly mediate its oncogenic activity. In contrast to other tumor types, the DEPDC1B‐mediated enhancement of melanoma metastatic potential is not dependent on the activities of RHO GTPase signaling and canonical Wnt signaling, but is acquired through secretion of signal peptide, CUB domain and EGF like domain containing 3 (SCUBE3), which is crucial for promoting angiogenesis in vitro and in vivo. Mechanistically, DEPDC1B regulates SCUBE3 protein stability through the competitive association with ubiquitin ligase cell division cycle 16 (CDC16) to prevent SCUBE3 from undergoing degradation via the ubiquitin‐proteasome pathway. Importantly, expression of SOX10, DEPDC1B, and SCUBE3 are positively correlated with microvessel density in the advanced stage of melanomas. In conclusion, it is revealed that a SOX10‐DEPDC1B‐SCUBE3 regulatory axis promotes melanoma angiogenesis and metastasis, which suggests that targeting secreted SCUBE3 can be a therapeutic strategy against metastatic melanoma.

## Graphical abstract

An oncogenic mechanism is unraveled by which DEPDC1B functions downstream of SOX10 to promote melanoma angiogenesis and metastasis. DEPDC1B competitively interacts with CDC16 in the cytosol to block the ubiquitination and degradation of SCUBE3, enabling stabilized SCUBE3 to be secreted from melanoma cells for blood vessel recruitment to facilitate their growth, survival, and metastasis.

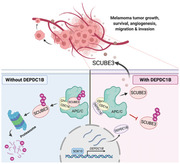

## 1 Introduction

Melanoma is one of the most devastating human cancers and is responsible for more than 80% of all skin cancer deaths.^[^ ^1^ ^]^ The aggressiveness of melanoma is due to the combination of oncogenic mutations (>50% of patients with BRAFV600E mutation) and dysregulated expression of cancer‐related genes that cause malignant transformation of neural crest‐derived melanocytes leading to metastatic melanoma.^[^ ^2^, ^3^ ^]^ Cutaneous melanoma is surgically curable in the early stages with survival rates of up to 98%, but survival drops to 27% once it undergoes metastasis and spreads to distant organs.^[^ ^4^ ^]^ The ability of the proliferative primary epidermal melanoma to migrate across the dermal stroma signifies the progression of melanoma from a primary to metastatic state, as defined by the Clark's staging system.^[^ ^5^ ^]^ This transition is driven by changes in the expression of oncogenes that enable metastatic melanoma to gain access to underlying blood vessels, where they secrete pro‐angiogenic factors to stimulate the formation of a vascular network from the existing blood vessels in a process called angiogenesis.^[^ ^6^ ^]^ Thus, increased blood vessel density in the tumor periphery is associated with metastasis and poor survival in melanoma patients.^[^ ^7^, ^8^ ^]^ Although current therapeutic options that target angiogenesis have been shown to have promising antitumor potential, acquired drug resistance compromises their efficacy.^[^ ^9^ ^]^ Moreover, melanoma heterogeneity implies the dysregulation of oncogene expression in a subset of melanomas, which can lead to the secretion of distinct pro‐angiogenic factors contributing to melanoma angiogenesis and metastasis. Therefore, the identification and characterization of more pro‐angiogenic factors and how they are regulated will provide alternative therapeutic targets against metastatic melanoma.

Dysregulation of DEPDC1B expression is frequently associated with oncogenesis and metastasis in different cancer types.^[^ ^10^, ^11^, ^12^, ^13^, ^14^, ^15^, ^16^, ^17^, ^18^ ^]^ For example, exogenous expression of DEPDC1B delays cell death and increases cell proliferation in breast cancer cells;^[^ ^19^ ^]^ overexpression of DEPDC1B enhances migration and invasion of non‐small‐cell lung cancer cells through the activation of Wnt/*β*‐catenin signaling;^[^ ^10^ ^]^ DEPDC1B promotes anchoring‐independent growth, migration, and invasion of oral cancer cells via the activation of Rac1‐ERK signaling axis;^[^ ^11^ ^]^ it also regulates the migration and invasion of pancreatic cancer through the activation of the RAC1/PAK1‐LIMK1‐cofilin1 signaling pathway;^[^ ^13^ ^]^ contributes to the migration and invasion of pancreatic ductal adenocarcinoma cells via the activation of Akt/GSK3*β*/Snail pathway;^[^ ^16^ ^]^ and is required for the development of glioblastoma^[^ ^14^ ^]^ and bladder cancer.^[^ ^17^ ^]^ Recent studies have shown that DEPDC1B also induces epithelial‐mesenchymal transition and promotes prostate cancer cell proliferation via Rac1‐PAK signaling.^[^ ^15^ ^]^ Altogether, these studies demonstrate context‐dependent roles of DEPDC1B in cancer growth and migration through the activation of different downstream events. A previous report also showed that DEPDC1B is required for melanoma growth and survival,^[^ ^20^ ^]^ but whether it has a role in melanoma metastasis remains to be determined.

SCUBE3 is a member of the SCUBE family of secreted glycoproteins, and its upregulation is positively correlated with the malignant development of several tumor types.^[^ ^21^, ^22^, ^23^, ^24^ ^]^ Moreover, SCUBE3 was found to regulate glioma cell proliferation.^[^ ^25^ ^]^ In addition, SCUBE3 promotes breast cancer progression through the activation of TGF‐*β*1 signaling.^[^ ^24^ ^]^ Similarly, SCUBE3 binds to TGF‐*β* type II receptor to trigger downstream signaling events involved in early lung cancer angiogenesis and metastatic progression.^[^ ^26^ ^]^ Whether SCUBE3 regulates melanoma growth, angiogenesis, and metastasis remains to be elucidated.

Here, we found that increased expression of *DEPDC1B* in cutaneous melanoma was significantly associated with a poor prognosis. Immunofluorescence analysis of a tissue microarray showed extensive overlap in the expression of SOX10, which is as key driver of melanoma initiation and progression,^[^ ^27^, ^28^ ^]^ and the expression of DEPDC1B in the different stages of melanoma. In agreement with this, both loss‐ and gain‐of‐function studies demonstrated that DEPDC1B was required and sufficient for melanoma growth, tumorigenicity, invasion and angiogenesis in vitro and in vivo. Chromatin immunoprecipitation, luciferase reporter and epistasis analyses revealed that DEPDC1B functions as a direct downstream target of SOX10 that partly mediates its oncogenic activity. In contrast to other cancer types, the ability of DEPDC1B to enhance the metastatic potential of melanoma does not dependent on the activities of Rho GTPase signaling and canonical Wnt signaling, but involves the secretion of SCUBE3, which was shown to promote angiogenesis in vitro and in vivo. The mechanistic studies both in vitro and in vivo showed that increased expression of DEPDC1B enhanced SCUBE3 protein stability through the competitive interaction with ubiquitin ligase CDC16, which prevented SCUBE3 from undergoing degradation via the ubiquitin‐proteasome pathway. The SOX10‐regulated DEPDC1B expression to stabilize SCUBE3 for promoting angiogenesis and metastasis was further supported by their high correlation of expression with microvessel density in metastatic melanoma specimens. Altogether, our findings reveal a SOX10‐DEPDC1B‐SCUBE3 regulatory axis that promotes melanoma angiogenesis and facilitates its metastatic progression, suggesting the possibility of targeting secreted SCUBE3 as a therapeutic strategy against metastatic melanoma.

## 2 Results

### 2.1 Elevated DEPDC1B Expression in Primary and Metastatic Melanomas

**Figure 1:**
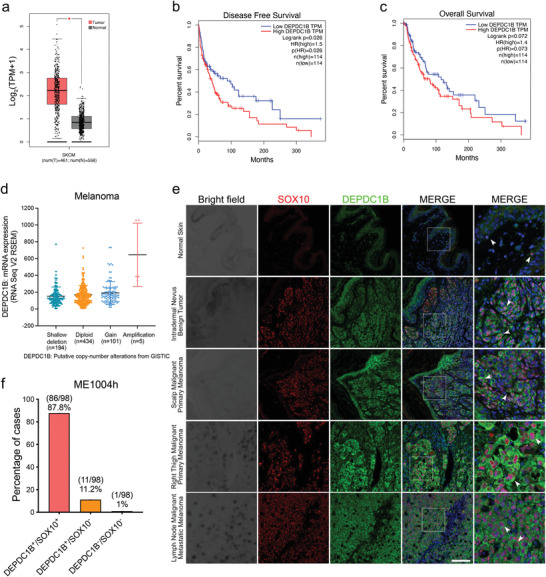
DEPDC1B expression level is elevated in melanoma patient samples. a) Gene expression profiling analysis of *DEPDC1B* mRNA levels in skin cutaneous melanoma (SKCM) tumor samples (red, *n* = 461) compared with normal skin tissues (grey, *n* = 558). Kaplan–Meier analysis of b) disease‐free survival and c) overall survival of 228 SKCM patients showing high *DEPDC1B* expression (75% cutoff) and low *DEPDC1B* expression (25% cutoff). d) TCGA analysis of *DEPDC1B* gene copy number variation in patients. e) Representative immunofluorescence (IF) images for SOX10 and DEPDC1B in normal skin, nevus and three melanoma specimens in the tissue array. White arrowheads indicate representative cells with colocalization of DEPDC1B and SOX10. Scale bar = 50 µm. f) Percentage of cases with DEPDC1B^+^/SOX10^+^, DEPDC1B^+^/SOX10^–^, and DEPDC1B^–^/SOX10^–^. This is amongst 62 cases of primary melanoma, 22 metastatic malignant melanoma, 14 nevus tissues, and 2 skin tissues.

Analysis of the TCGA dataset revealed a substantial portion of melanoma patients exhibited high levels of *DEPDC1B* transcripts in cutaneous melanoma tissues (*n* = 461) compared with normal tissues (*n* = 558) (**Figure** **1**a). Kaplan–Meier analysis showed that high *DEPDC1B* expression was significantly associated with decreased disease‐free survival and overall survival compared with the cohort with low levels of *DEPDC1B* transcripts (Figure 1b,c). However, only a few melanoma specimens (*n* = 5) showed *DEPDC1B* amplification and most (*n* = 434) were diploid, suggesting other factors contributed to the increase in *DEPDC1B* transcription (Figure 1d). To further examine DEPDC1B protein expression in melanoma tissue samples, we performed immunofluorescence staining in a melanoma tissue microarray (TMA), which contained 62 cases of primary melanoma, 22 metastatic malignant melanoma, 14 nevus tissues, and two skin tissues. We detected overlapping expressions of cytoplasmic DEPDC1B and SOX10 transcription factor in a large percentage of nevus, primary, and metastatic melanomas (86/98; 87.8%) compared with specimens expressing DEPDC1B alone (11/98; 11.2%) or expressing neither protein (1/98; 1%) (Figure 1e,f). In contrast, expressions of DEPDC1B and SOX10 were weak and barely detectable in normal skin tissue, respectively (Figure 1e). These results coincide with the TCGA analysis that elevated *DEPDC1B* expression is associated with primary and metastatic melanomas.

### 2.2 DEPDC1B Promotes Melanoma Metastasis and Angiogenesis

**Figure 2:**
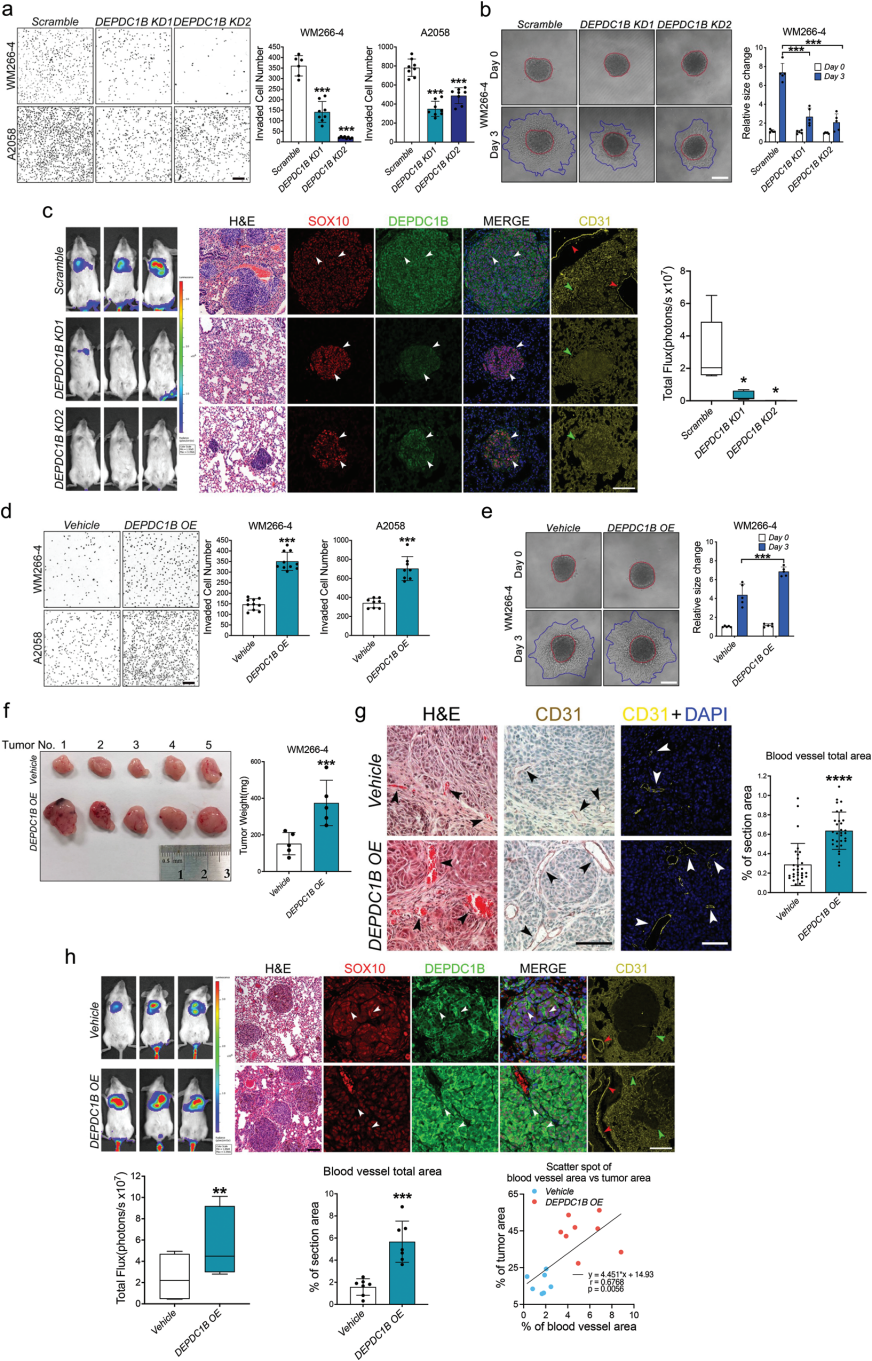
DEPDC1B promotes melanoma growth, invasion, metastasis and angiogenesis. a) Representative images of transwell‐invaded cells treated with control (*scramble*), *DEPDC1B KD* (*DEPDC1B KD1* and *DEPDC1B KD2*). Cell number was counted using ImageJ software, *n* ≥ 6. Scale bar = 200 µm. b) Representative images showing 3D spheroid invasion into collagen matrix. Red dotted lines mark the spheriods' margin on embedding day (Day 0) and blue dotted lines mark the invaded spheroids' margin 3 d after embedding (Day 3). Pixel analysis of images was performed to calculate the total area occupied by the tumor spheroids, *n* = 5. Scale bar = 100 µm. c) In vivo bioluminescence of metastatic lungs with indicated treatments and hematological & eosin (H&E) staining of sections from lung nodules, *n* = 5. Scale bar = 100 µm. The IF staining of paraffin wax‐embedded mice lung sections was performed against SOX10, DEPDC1B and CD31. The nuclei were counterstained with DAPI. White arrowheads mark representative signals, green arrowheads mark the position of nodules and red arrowheads mark the position of microvessels. Scale bar = 50 µm. d) Representative images of transwell‐invaded cells treated with control (*vehicle*), *DEPDC1B OE*. *n* ≥ 7. Scale bar = 200 µm. e) Representative images showing 3D spheroid invasion into collagen matrix. *n* = 5. Scale bar = 100 µm. f) Image of xenografts from WM266‐4 cells infected by the indicated constructs, *n* = 5. Tumor weights were measured and analyzed as shown. g) The subcutaneous tumors were sectioned and quantified for the blood vessel formation based on the IF staining against CD31. Two tumors from each group were sectioned and three adjacent sections were chosen for H&E staining, fluorescent‐IHC staining and DAB‐IHC staining respectively. Triplicates were obtained every 100 µm apart and five random views were imaged in each section for quantification by Image J. *n* = 2×3×5 = 30. Scale bar = 100 µm. h) In vivo lung metastasis of WM266‐4 cells with control (*vehicle*), *DEPDC1B OE*. White arrowheads indicate representative signals, green arrowheads mark the position of nodules and red arrowheads mark the position of microvessels. *n* = 7. The % of tumor area and the % of blood vessel area were correlated by simple linear regression. Scale bar = 50 µm. Data are mean ± standard derivation (SD). The bioluminescence data are present from minimal to maximum. *p < 0.05; **p < 0.01; ***p < 0.001; ****p < 0.0001 from unpaired Student′s t‐test, ordinary one‐way ANOVA and two‐way ANOVA.

Previous studies using a single shRNA demonstrated that DEPDC1B is required for melanoma proliferation and tumorigenicity.^[^ ^20^ ^]^ To consolidate the findings, we used two different shRNA lentiviral constructs to stably silence *DEPDC1B* transcripts (*KD1, KD2*) in BRAF‐mutated WM266‐4, A2058, and SK‐MEL‐28 melanoma cell lines and obtained similar outcomes with a marked reduction of proliferation, colony formation and subcutaneous tumor growth compared with controls (Figure S1a–d, g, Supporting Information). We further extended the analysis on cell invasion using transwell and spheroid migration assays as well as lung colonization assay in NOD/SCID mice. Both *DEPDC1B KD1* and *KD2* cells showed reduced invasiveness and lung metastases (**Figure** **2**a–c, Figure S1e,f,h, Supporting Information). In contrast, lentiviral‐mediated overexpression of DEPDC1B (*DEPDC1B OE*) significantly promoted cell proliferation (Figure S2a–d, Supporting Information), colony formation (Figure S2e, Supporting Information), and invasion (Figure 2d,e, Figure S2f,g, Supporting Information) compared with the vehicle control. Accordingly, subcutaneous and tail vein injection of *DEPDC1B OE* cells in immunodeficient mice resulted in enhanced tumor growth and lung colonization (Figure 2f–h, Figure S2h,i, Supporting Information). Notably, histological and immunohistological analysis of sections from tumor xenografts showed *DEPDC1B OE* promoted the formation of CD31^+^ microvessel with lumens containing red blood cells in the periphery compared to the vehicle control (Figure 2g), suggesting an enhancement of angiogenesis by high level of DEPDC1B expression. To further investigate the impact of *DEPDC1B KD* and OE on angiogenesis in vivo, we performed immunofluorescence for SOX10, DEPDC1B and CD31 on sections from lung nodules of tail vein injected mice. Expression of DEPDC1B and SOX10 were detected in most of the control cells (Figure 2c), as was observed in patient samples (Figure 1e,f), whereas DEPDC1B expression was markedly reduced in SOX10^+^ residual tissue derived from *DEPDC1B KD* cells (Figure 2c). Moreover, a notable reduction of CD31^+^ microvessel formation in the periphery was observed in *DEPDC1B KD* compared with controls (Figure 2c). Conversely, elevated expression levels of DEPDC1B were detected in SOX10‐expressing cells within larger‐sized lung nodules with *DEPDC1B OE* compared with moderate expression levels of DEPDC1B in nodules with the vehicle control (Figure 2h). In addition, *DEPDC1B OE* resulted in a higher peripheral microvessel density positively correlated with the size of lung nodules (Figure 2h). Taken together, these data demonstrate the oncogenic role of DEPDC1B in promoting melanoma metastasis and angiogenesis.

### 2.3 DEPDC1B Functions Downstream of SOX10 to Partly Mediate Its Oncogenic Activity

**Figure 3:**
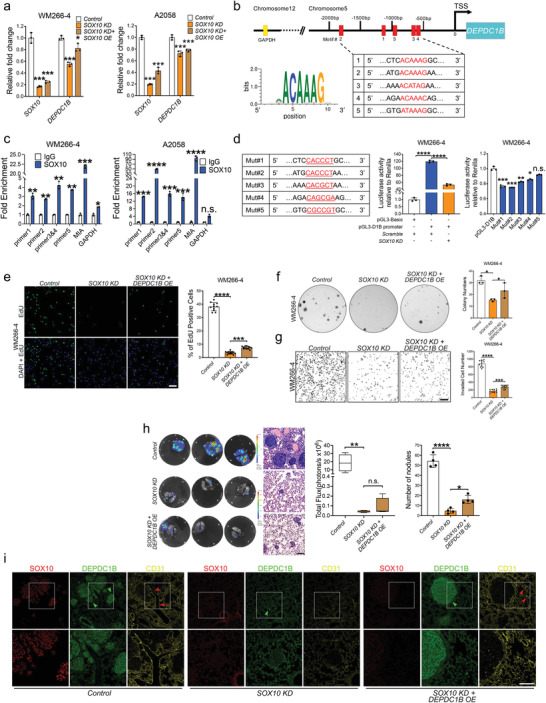
DEPDC1B functions downstream of SOX10 to partly mediate its oncogenic role. a) qPCR analysis of *SOX10* and *DEPDC1B* mRNA levels in melanoma cells treated with *control* (*scramble*), *SOX10 KD* alone, and *SOX10 KD + DEPDC1B OE*, *n* = 3. b) Schematic diagram showing the genomic location of *DEPDC1B* regulatory regions containing *SOX10* binding motifs naming in a 5’ to 3’ order as motifs 2, 1, 5, 3 and 4 based on their degree of similarity to a SOX10 consensus sequence as evaluated by the JASPAR database. c) ChIP‐qPCR analysis showing fold enrichment levels of motifs bound by SOX10 compared with IgG control, *n* = 3. d) The wild‐type *DEPDC1B* promoter (‐2071bp to ‐57bp) was cloned into pGL3‐based vector and the activity was evaluated in WM266‐4 cells treated with *SOX10 KD* or scramble control by dual luciferase reporter assay. The six nucleotides within the five SOX10 consensus motifs were mutated (A/T to C/G, and vice versa) and each mutated promoter activity was compared with the wild‐type promoter by luciferase reporter assay. e) *DEPDC1B OE* in *SOX10 KD* partially restored the number of EdU^+^ cells (*n* ≥ 7), f) colony formation (*n* = 3), and g) transwell‐invaded cells (*n* ≥ 7) compared with *SOX10 KD* alone. Scale bar = 200 µm. h) Bioluminescence analysis showed a limited degree of rescue in lung metastases by *DEPDC1B OE* in *SOX10 KD* cells compared with *SOX10 KD* alone and parental control, *n* = 4. H&E staining of the corresponding lung sections. Due to the strong inhibitory effect of *SOX10 KD* on lung metastasis, the level of bioluminescence emitted by the residual number of nodules from the rescue group might not be strong enough to discern the significant difference between *SOX10 KD* group and *SOX10 KD* + *DEPDC1B OE* group. In contrast, there was a significant increase in the number of lung nodules formed in the rescue group compared with the *SOX10 KD* group. i) IF staining of mice lung sections against SOX10, DEPDC1B, and CD31. Green arrowheads indicate tumor nodules and red arrowheads mark the position of microvessels. Scale bar = 50 µm. Data are mean ± SD. The bioluminescence data are present from minimal to maximum. n.s. (not significant) when *p* > 0.05; *p < 0.05; **p < 0.01; ***p < 0.001 from ordinary one‐way ANOVA and two‐way ANOVA.

The overlapping expressions of SOX10 and DEPDC1B in patient samples and in the tumor nodules prompted us to investigate whether SOX10 regulates DEPDC1B expression. Indeed, *SOX10 KD* significantly reduced the level of *DEPDC1B* expression compared with controls, whereas *SOX10 OE* moderately restored *DEPDC1B* expression in *SOX10*‐depleted cells (**Figure** **3**a, Figure S3a, Supporting Information). In silico analysis identified five SOX10 consensus binding motifs within 2 kb of the *DEPDC1B* promoter region (Figure 3b). Chromatin immunoprecipitation (ChIP) assay confirmed that endogenous SOX10 had stronger binding abilities for these motifs in A2058 than in WM266‐4 (Figure 3c, Figure S3b,c, supporting information). It is possible that distinct epigenetic regulation at the *DEPDC1B* promoter and availability of SOX10 cofactors in different cell lines could modulate SOX10 binding capacities to different motifs as evidenced by previous studies in other cellular contexts.^[^ ^29^, ^30^, ^31^ ^]^ Notably, the ChIP signals of SOX10 binding to *DEPDC1B* promoter in both cell lines were much weaker than that to MIA positive control promoter which has been shown to bind SOX10 in melanoma cell lines^[^ ^32^ ^]^ (Figure 3c), implying distinct binding capacities of SOX10 on different target gene promoters. To evaluate the transactivation ability of SOX10 on *DEPDC1B* promoter, we performed luciferase reporter assay with a wild‐type *DEPDC1B* promoter and 5 constructs with each containing multiple point mutations within the *DEPDC1B* promoter (Figure 3b,d). *SOX10 KD* caused a more than twofold reduction of wild‐type reporter activity compared with scramble control (Figure 3d, Figure S3c, Supporting Information). Mut#1, 2 or 3 led to around 20% reduction in the reporter activity compared to wild‐type promoter. In contrast, Mut#4 and #5 showed subtle or negligible reduction in the promoter activities though ChIP signals can be detected in these motifs (Figure 3b,d, Figure S3c, Supporting Information). As motifs 3 and 4 as well as motifs 1 and 5 are spaced apart by 29 bp and 129 bp respectively (Figure 3b), it is likely that they were enriched together by SOX10. Altogether, these results suggest that SOX10 promotes *DEPDC1B* transcription through binding to motifs 2, 1 and 3 in the *DEPDC1B* promoter.

To examine whether DEPDC1B functions downstream of SOX10 to regulate melanoma growth and metastasis, we performed an epistasis analysis. Treatment of *SOX10 KD* cells with *DEPDC1B OE* resulted in the partial rescue of melanoma cell proliferation (Figure 3e, Figure S3d, Supporting Information), colony formation (Figure 3f, Figure S3e, Supporting Information), invasion (Figure 3g, Figure S3f, Supporting Information), lung metastasis (Figure 3h, Figure S3g, Supporting Information), and CD31^+^ microvessel formation in lung nodules (Figure 3i) compared with *SOX10 KD* alone. These results suggest that DEPDC1B partly mediates the role of SOX10 in melanoma growth, metastasis, and angiogenesis.

### 2.4 DEPDC1B‐Mediated Melanoma Metastasis Is Not Dependent on Canonical Wnt Signaling and Rho GTPase Signaling Activities

As previous studies showed that DEPDC1B enhanced migration and invasion of non‐small‐cell lung cancer through the activation of canonical Wnt/*β*‐catenin pathway,^[^ ^10^ ^]^ we investigated whether a similar phenomenon occurs in melanoma cells. To this end, we used TOPflash reporter to measure the transcriptional activity of nuclear *β*‐catenin as a readout for canonical Wnt signaling in three melanoma cell lines treated with *DEPDC1B KD* or *OE* (Figure S4a–c, Supporting Information). The results showed that neither *DEPDC1B KD* nor *OE* significantly altered the reporter activity compared with the scramble control. Supporting this finding, the immunofluorescence assay did not detect altered subcellular localization of *β*‐catenin (Figure S4d, Supporting Information). As DEPDC1B contains a RhoGAP domain that is also functionally important in cytoskeletal remodeling in cell motility,^[^ ^33^ ^]^ we examined whether Rho GTPase signaling is also involved. We focused on RHOA and RAC1, whose activities have been shown to be regulated by DEPDC1B in different cellular contexts.^[^ ^11^, ^34^, ^35^ ^]^ We used RHOA and RAC1 activation assays to examine the impact of *DEPDC1B KD* and *OE* on the levels of the active forms of RHOA‐GTP and RAC1‐GTP in melanoma cells, respectively. The results showed that neither *DEPDC1B KD* nor *OE* markedly altered RHOA‐GTP and RAC1‐GTP levels compared with their respective controls (Figure S4e–h, Supporting Information). In agreement with this, ERK activity was not altered (Figure S4i,j, Supporting Information). This differs from previous studies that showed DEPDC1B activated ERK activity through RAC1 to promote anchorage‐independent growth in oral cancer cells.^[^ ^11^ ^]^ Altogether, our results showed that DEPDC1B promotes melanoma metastasis independent of canonical Wnt signaling and Rho GTPase signaling activities, which suggests other molecules are involved.

### 2.5 DEPDC1B Regulates the Expression of Secreted SCUBE3 Protein

**Figure 4:**
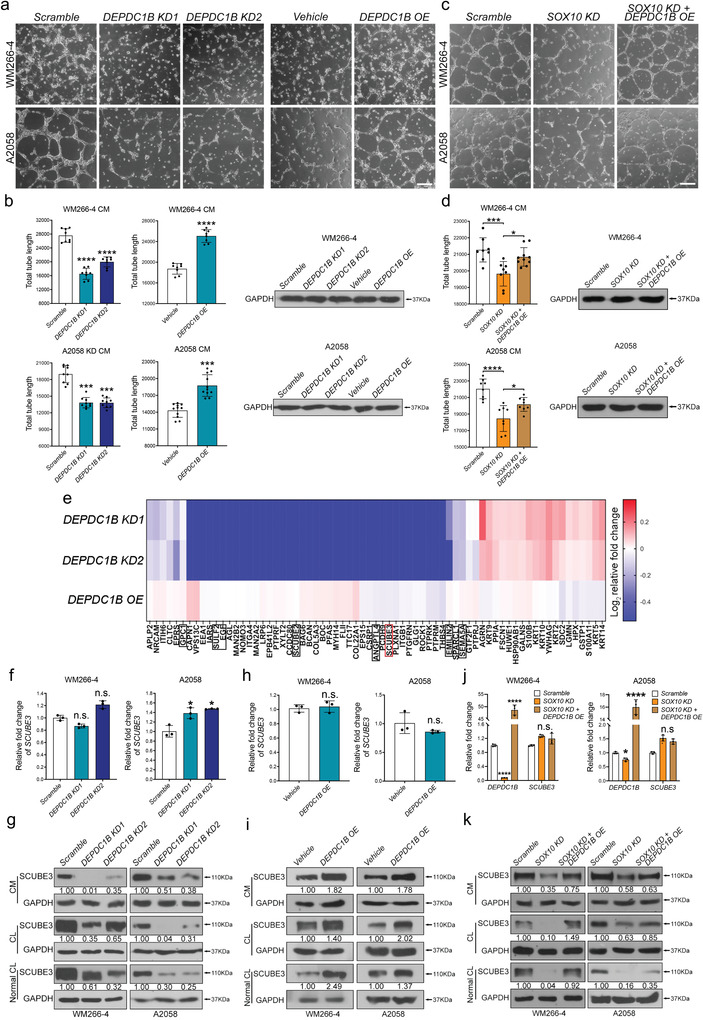
DEPDC1B is required for angiogenesis by regulating the expression of secreted SCUBE3 protein. a) Representative images of HUVECs cultured with CM from WM266‐4 (upper panel) and A2058 (lower panel) cells treated with *scramble*, *DEPDC1B KD1 & KD2*, *vehicle*, and *DEPDC1B OE*. b) Images were taken after incubation for 7–9 h, and 6–10 random views were imaged and quantified using ImageJ. Western blot analysis of cell lysates for GAPDH indicating equal cell confluency between samples. Scale bar = 200 µm. c) Representative images of HUVECs cultured with CM from WM266‐4 (upper panel) and A2058 (lower panel) cells treated with *scramble*, *SOX10 KD*, and *SOX10KD* + *DEPDC1B OE*. Scale bar = 200 µm. d) Quantification of total tube lengths formed by HUVECs in different treatments. Western blot analysis of cell lysates for GAPDH showing equal cell confluency. e) CM collected from WM266‐4 cells treated with *scramble*, *DEPDC1B KD1*, *KD2*, *vehicle*, and *DEPDC1B OE* were subjected to Mass Spectrometry analysis. Heat map showing protein abundance differences. All values were normalized with scramble and vehicle control, respectively. The protein abundance was set to 0.001 when the protein was not detected instead of NA for normalization convenience. f,h) qPCR analysis of *SCUBE3* mRNA levels in melanoma cells treated with *DEPDC1B KD* and OE, *n* = 3. g,i) Western blot analysis verified changes in SCUBE3 protein levels with the indicated treatments. j,k) qPCR and Western blot analysis of the impact of *SOX10 KD* and *DEPDC1B OE* rescue on SCUBE3 expression. CM: conditioned medium. CL: lysate of the remaining cells after CM collection. Normal CL: Lysate of cells that did not undergo serum‐starvation (i.e., CM collection). Data are mean ± SD. n.s. when *p* > 0.05; *p < 0.05; **p < 0.01; ***p < 0.001; ****p < 0.0001 from unpaired Student′s *t*‐test, ordinary one‐way ANOVA and two‐way ANOVA.

In the melanoma pulmonary colonization assay, one notable observation was the respective reduction and increase of peripheral microvessel density in lung nodules following treatment with *DEPDC1B KD* and *OE*, indicating DEPDC1B can induce the expression of secreted pro‐angiogenic factors to promote angiogenesis and metastasis. To this end, we further evaluated the effects of the conditioned media (CM) from *DEPDC1B KD* and *OE* cells on angiogenesis in human umbilical vein endothelial cells (HUVECs). After 7–9 h incubation of HUVECs in CM from cells treated with scramble control or vehicle control, tube‐like structures harboring branches, segments and nodes were observed (**Figure** **4**a, Figures S5a and S6a, Supporting Information). In contrast, incubation of HUVECs with CM from *DEPDC1B KD* and *OE* cells significantly inhibited and increased the total length, segments and branches of the tubular network, respectively (Figure 4a,b, Figures S5a and S6a,g, Supporting Information). In addition, *DEPDC1B OE*+*SOX10 KD*‐CM partly restored the formation of the HUVEC tubular network compared with a marked reduction of the total tube length, segments and branches in *SOX10 KD*‐CM treatment alone (Figure 4c,d, Figures S5b and S6b,h, Supporting Information), further confirming that DEPDC1B partly mediates the role of SOX10 in angiogenesis. These results further suggested the presence of pro‐angiogenic factors in the CM secreted by DEPDC1B‐expressing melanoma cells. To identify these pro‐angiogenic factors, we performed mass spectrometry of the CM from *DEPDC1B KD* and *OE* cells (Tables S1 and S2, Supporting Information). Among the differentially expressed secreted proteins between the two treatments, we selected SCUBE3 for further analysis, as it has been shown to regulate early lung cancer angiogenesis and metastasis (Figure 4e).^[^ ^26^ ^]^ In addition, we found DEPDC1B was predominantly localized in the cytoplasm of melanoma specimens (Figure 1e), indicating it does not have a role in regulating gene transcription. At the transcriptional level, *SCUBE3* gene was among the least affected by *DEPDC1B KD* and *OE* (Figure 4f,h, Figure S5c,d, Supporting Information). Although we detected a slight increase in the amount of *SCUBE3* mRNA in A2058 cells treated with *DEPDC1B KD* (Figure 4f, Figure S5d, Supporting Information), the protein expression level of SCUBE3 was markedly reduced in the CM and whole cell lysates from both *DEPDC1B KD* WM266‐4 and A2058 cell lines (Figure 4g). In contrast, *DEPDC1B OE* increased the amount of endogenous SCUBE3 protein (Figure 4i). However, SCUBE3 was not detected in less aggressive melanoma SK‐MEL‐28 cells (Figure S5e, Supporting Information). Moreover, *SOX10 KD* did not affect *SCUBE3* mRNA, but reduced SCUBE3 protein expression level, which was partly restored by *DEPDC1B OE* (Figure 4j,k). These results suggest that DEPDC1B functions downstream of SOX10 in regulating the expression of secreted SCUBE3 protein.

### 2.6 SCUBE3 Secretion Promotes Angiogenesis and Melanoma Progression

**Figure 5:**
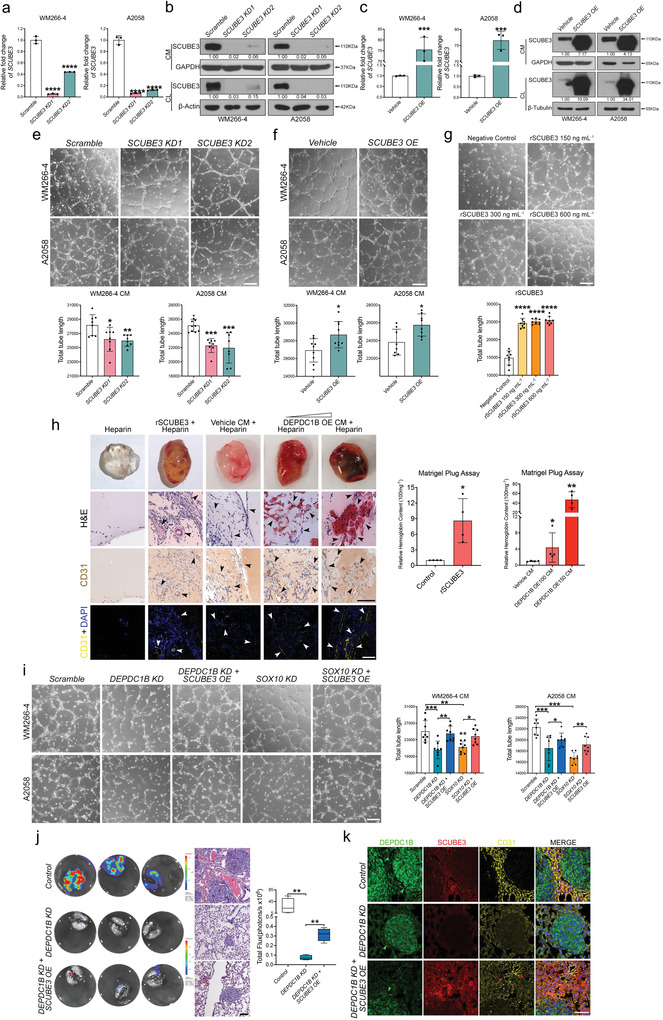
SCUBE3 is pro‐angiogenic and can partially restore the depleted metastasis of *DEPDC1B KD*. a,c) qPCR analysis to validate the efficiency of *SCUBE3* knock‐down (*SCUBE3 KD1 and SCUBE3 KD2*) and SCUBE3 overexpression (*SCUBE3 OE*) at the mRNA level, *n* = 3. b,d) Western blot analysis to validate *SCUBE3 KD* and *OE* at the protein level. e) Representative images of in vitro HUVEC tube formation assay cultured with *SCUBE3 KDs* CM collected from WM266‐4 and A2058 cells. f) HUVECs were cultured with *SCUBE3 OE* CM collected from both cell lines. g) Recombinant SCUBE3 (rSCUBE3) protein at different doses was added into non‐supplemented M200 HUVEC culture medium (Negative Control). 8–9 random views were quantified for total tube length. h) Representative images of the implanted Matrigel plugs containing heparin (negative control), heparin + rSCUBE3 or heparin mixed with concentrated WM266‐4 CM from vehicle control, 100 and 150 µL lentiviruses expressing DEPDC1B. The hemoglobin content level was measured using Drabkin's Reagent. Representative images are H&E staining and IHC, IF staining against CD31. Arrowheads mark the CD31^+^ microvessels. Scale bar = 100 µm. i) Restoration of tube formation capacity by *SCUBE3 OE* in *SOX10 KD* and *DEPDC1B KD* CM. All images were taken 7–9 h after incubation with the indicated CM. 8 random views were imaged and quantified. j) Bioluminescence of metastatic lungs with the indicated treatments and H&E staining of their corresponding sections. *n* = 4. Scale bar = 100 µm. k) IF staining of paraffin wax‐embedded mice lung sections against DEPDC1B, SCUBE3, and CD31. The nuclei were counterstained with DAPI. Green arrowheads mark the position of nodules and red arrowheads mark the position of microvessels. Scale bar = 50 µm. Data are mean ± SD. The bioluminescence data are present from minimal to maximum. *p < 0.05; **p < 0.01; ***p < 0.001; ****p < 0.0001 from unpaired Student′s *t*‐test and ordinary one‐way ANOVA.

To further investigate whether SCUBE3 secreted from melanoma cells contributes to angiogenesis, we designed two shRNAs to deplete *SCUBE3 mRNA* (*SCUBE3 KD1* and *KD2*) and proteins (**Figure** **5**a,b), and generated lentiviral vector‐mediated *SCUBE3* overexpression (*SCUBE3 OE*) in both WM266‐4 and A2058 cells (Figure 5c,d). The results showed that HUVECs treated with CM from *SCUBE3 KD* and *OE* cells resulted in a marked reduction and increase of the tubular network, respectively (Figure 5e,f, Figure S6c,d, Supporting Information). In addition, CM from *SCUBE3 OE* SK‐MEL‐28 cells also led to an increase in the total tube length, segments and branches of the network (Figures S5f and S6i, Supporting Information). To further evaluate the secretory role of SCUBE3 in angiogenesis, HUVECs were treated with different dosages of human recombinant SCUBE3 protein, which resulted in a considerable increase in the tube length with 150 ng mL^–1^ SCUBE3 compared with nonsupplemented M200 HUVEC CM control. No further increases in the length of the tubular networks were observed with 300 or 600 ng mL^–1^ SCUBE3 (Figure 5g, Figure S6e, Supporting Information), implying a saturated response in HUVECs at the lower dosage of recombinant SCUBE3 protein. Similar enhancements on the tubular network formation were observed with 600 ng mL^–1^ recombinant SCUBE3 in CM from melanoma parental cells (Figures S5g,h and S6j,k, Supporting Information). To further demonstrate the in vivo role of SCUBE3 in angiogenesis, we performed the Matrigel plug assay in wild‐type mice. The plugs recovered from mice 7 d post‐implantation showed that recombinant SCUBE3 protein stimulated angiogenesis as shown by an increase in hemoglobin level compared to control Matrigel which contained heparin alone and exhibited low level of hemoglobin in trace of vessels (Figure 5h). Accordingly, hematoxylin and eosin staining revealed that recombinant SCUBE3 protein induced a strong angiogenic response with an increased number of CD31^+^ vascular structures with lumens and red blood cells as compared with the control plugs (Figure 5h). Similarly, we observed intense vascularization in plugs containing CM from different volume of *DEPDC1B OE* lentiviruses. Hemoglobin content was higher in the plugs containing CM from 150 µL *DEPDC1B OE* lentivirus than in the plug containing CM from 100 µL *DEPDC1B OE* lentivirus and from vehicle control (Figure 5h), indicating a dose‐dependent angiogenic response. These results demonstrate the pro‐angiogenic role of SCUBE3 and DEPDC1B in vivo. In addition, CM from *DEPDC1B KD*+*SCUBE3 OE* and *SOX10 KD*+*SCUBE3 OE* cells restored the length, branches and segments of tubular networks to a greater extent compared with the marked reduction in their formation with CM from cells treated with *DEPDC1B KD* and *SOX10 KD* alone, respectively (Figure 5i, Figure S6f, Supporting Information). Accordingly, *SCUBE3 OE* partially restored the lung colonization capacity of *DEPDC1B KD* cells (Figure 5j, Figure S5i, Supporting Information) and was accompanied by a high peripheral microvessel density in lung nodules compared with *DEPDC1B KD* alone (Figure 5k). These findings suggest that SCUBE3 secretion mediates SOX10 and DEPDC1B in promoting melanoma angiogenesis and metastasis. In addition, melanoma cells treated with *SCUBE3 KD1* and *KD2* exhibited marked reductions in proliferation, number of colonies formed, and invasive capacity compared with the scramble control (Figure S5j–l, Supporting Information), which further consolidates the oncogenic role of SCUBE3 in melanoma.

### 2.7 DEPDC1B Regulates SCUBE3 Protein Stability through Sequestration of Ubiquitin Ligase CDC16

**Figure 6:**
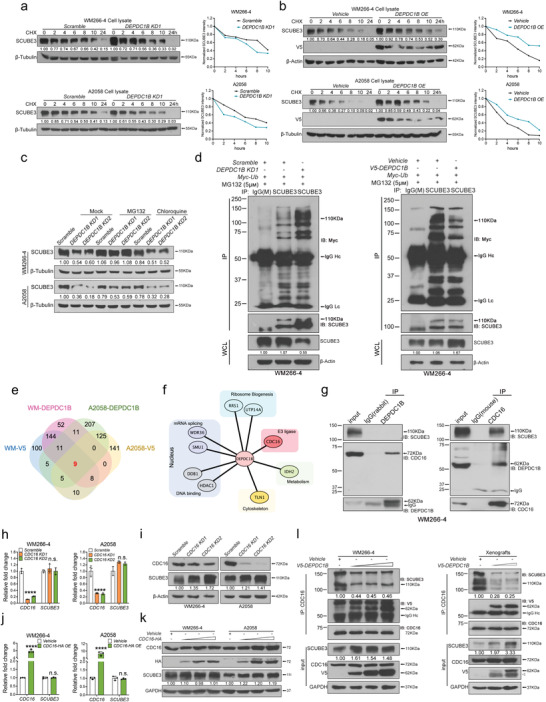
DEPDC1B stabilizes SCUBE3 by competitive association with CDC16, inhibiting the ubiquitylation and degradation of SCUBE3. a,b) Melanoma cells were infected with the indicated treatments for 72 h then treated with cycloheximide (CHX) for different incubation periods and then protein degradation rate was evaluated. Protein bands from Western blots were quantified using Image J. Two independent replicates were performed on the CHX chase assay. c) The proteasome inhibitor, MG132, was applied to WM266‐4 (5 × 10^−6^ m, 24 h) and A2058 (20 × 10^−6^ m, 6 h) cells. The autophagy inhibitor, Chloroquine, was applied to both cell lines (100 × 10^−6^ m, 24 h). d) WM266‐4 cells were transduced with DEPDC1B and Myc‐Ubiquitin for 72 h followed by MG132 (5 × 10^−6^ m) treatment for 6 h prior to SCUBE3 IP. The amount of cell lysate used for IP was normalized proportionally to SCUBE3 whole cell lysate protein level. Anti‐Myc antibody was used to detect the ubiquitylated level of SCUBE3. e) Venn diagram showing the common proteins pulled down by endogenous and exogenous expression of DEPDC1B in both WM266‐4 and A2058 cell lines. IgG was used as the negative control (not shown) and proteins pulled down by IgG were excluded in the analysis. f) DEPDC1B interactome of the 9 common pull‐down candidates. g) Endogenous co‐immunoprecipitation using anti‐DEPDC1B antibody and anti‐CDC16 antibody. h,j) qPCR analysis showing the relative mRNA levels of CDC16 and SCUBE3 upon *CDC16 KDs* and *OE*, *n* = 3. i,k) Western blot analysis of lysates from CDC16 KDs and OE for SCUBE3 expression. l) Competitive co‐IP assays with endogenous CDC16 followed by Western blotting with DEPDC1B and SCUBE3 were conducted in WM 266‐4 cells and xenografts. *DEPDC1B* was over‐expressed in a dose gradient in WM266‐4 cells (left) for co‐IP assay. WM266‐4 cells transduced with vehicle control and two graded doses of lentiviruses expressing DEPDC1B were injected subcutaneously in nude mice (right). After four weeks, the tumors were dissected, grinded and lysed for the co‐IP assay. Lc and Hc indicate light chain and heavy chain of the antibody respectively. Data are mean ± SD. n.s. when *p* > 0.05; *p < 0.05; **p < 0.01; ***p < 0.001; ****p < 0.0001 from two‐way ANOVA.

In *DEPDC1B KD* cells, the reduction of SCUBE3 protein expression instead of mRNA level raises the possibility that DEPDC1B regulates SCUBE3 protein stability. To address this issue, *DEPDC1B KD* and *DEPDC1B OE* cells were treated with a well‐known inhibitor of protein biosynthesis cycloheximide (CHX) for different incubation periods to determine the effects on SCUBE3 half‐life. The CHX chase analysis showed that *DEPDC1B KD* treatment resulted in a shorter SCUBE3 half‐life in WM266‐4 and A2058 cells compared with their respective scramble controls (**Figure** **6**a, Figure S7a,b, Supporting Information). In contrast, *DEPDC1B OE* treatment resulted in a longer SCUBE3 half‐life in WM266‐4 and A2058 cells compared with the vehicle controls (Figure 6b). To further investigate the pathway involved in regulating SCUBE3 stability, *DEPDC1B KD* cells were treated with a common 26S proteasome inhibitor MG132 and an autophagy inhibitor chloroquine. The results showed that MG132 treatment efficiently restored SCUBE3 protein expression in *DEPDC1B KD* cells to a level similar to that in the scramble control. However, *DEPDC1B KD* cells treated with chloroquine did not restore the low SCUBE3 expression levels (Figure 6c). These results suggest that SCUBE3 destabilization is likely mediated by the ubiquitin‐proteosome pathway in *DEPDC1B KD* cells. Indeed, elevated and reduced levels of ubiquitylated SCUBE3 were detected in *DEPDC1B KD* and *OE* cells, respectively, compared with scramble and vehicle controls (Figure 6d, Figure S7c, Supporting Information), suggesting that DEPDC1B inhibits the ubiquitin‐dependent degradation of SCUBE3. To further elucidate the molecular mechanism by which DEPDC1B regulates SCUBE3 stability, we performed immunoprecipitation (IP) on endogenous and ectopic expressions of DEPDC1B in lysates extracted from both WM266‐4 and A2058 cells followed by mass spectrometry (Figure 6e, Tables S3 and S4, Supporting Information). Among all the protein candidates, only nine factors were enriched by DEPDC1B in all samples, of which CDC16 plays a role in regulating protein stability as part of the anaphase‐promoting complex/cyclosome (APC/C) E3 ubiquitin ligase (Figure 6f).^[^ ^36^ ^]^ Immunoprecipitation studies confirmed endogenous and ectopic interactions between DEPDC1B and CDC16 (Figure 6g, Figure S7d–h, Supporting Information), as well as between SCUBE3 and CDC16 in both cell lines (Figure 6g, Figure S7i–l, Supporting Information). However, there was no interaction between DEPDC1B and SCUBE3 (Figure 6g, Figure S7d, Supporting Information). To further map the domain of CDC16 interacting with DEPDC1B and SCUBE3, we generated two truncated CDC16 constructs (N1‐266 and N262‐620) (Figure S8a, Supporting information), but found neither was able to interact with ectopic DEPDC1B or endogenous SCUBE3 (Figure S8b–e, Supporting information), indicating the full‐length CDC16 protein is crucial for these interactions.

Moreover, *CDC16 KD* enhanced SCUBE3 protein levels without altering mRNA expression, confirming its functional requirement for regulating SCUBE3 stability (Figure 6h,i). In contrast, increasing the titer of lentiviral vector encoding CDC16 did not affect SCUBE3 mRNA or protein expression levels (Figure 6j,k). This could be due to the limited endogenous availability of anaphase‐promoting components to form a functional complex with excess CDC16 for protein degradation. The ability of CDC16 to interact with DEPDC1B and SCUBE3 prompted us to presume that DEPDC1B might stabilize SCUBE3 through competitive binding of CDC16. To validate this hypothesis, melanoma cells were transduced with increasing titers of lentiviruses expressing DEPDC1B followed by immunoprecipitation with endogenous CDC16, and the immune‐complexes were then analyzed by Western blotting for SCUBE3. Overexpression of DEPDC1B reduced the degree of CDC16 binding with SCUBE3, but not in a dose‐dependent manner compared with the vehicle control. Consistently, elevated levels of SCUBE3 protein were detected in DEPDC1B overexpressing cells (Figure 6l, Figure S8f, Supporting Information). Similar results were obtained using protein lysates from xenografts expressing graded levels of DEPDC1B protein (Figure 6l, Figure S8g, Supporting Information). Altogether, both in vitro and in vivo studies suggest that DEPDC1B stabilizes SCUBE3 by inhibiting its interaction with CDC16 to prevent subsequent ubiquitin‐dependent degradation.

### 2.8 Clinical Correlation between DEPDC1B and SCUBE3 in Melanoma Angiogenesis

**Figure 7:**
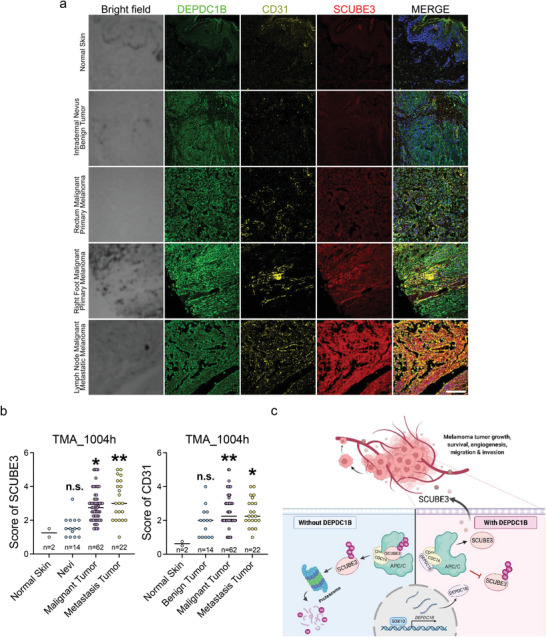
SCUBE3 has a higher expression level in metastatic melanomas accompanied by denser blood vessel distribution. a) TMA immunofluorescence staining using anti‐SCUBE3 (red), anti‐DEPDC1B (green), and anti‐CD31 (yellow) together with DAPI (blue in the merge) for nuclei counterstaining. Scale bar = 50 µm. b) Quantitative analysis of the SCUBE3 protein level and micro vessel distribution (marked by CD31) in different stages of melanoma. c) Schematic diagram shows SOX10 directly transactivates DEPDC1B expression through binding to its promoter region. DEPDC1B competitively interacts with CDC16 in the cytosol to inhibit SCUBE3 ubiquitination. Stabilized SCUBE3 secreted from melanoma cells facilitates blood vessel recruitment to promote melanoma tumor growth, survival, and metastasis. Data are median with individual values. n.s. when *p* > 0.05; *p < 0.05; **p < 0.01; ***p < 0.001; ****p < 0.0001 from ordinary one‐way ANOVA.

To establish the clinical relevance of DEPDC1B and SCUBE3 on angiogenesis, we analyzed their expression in association with CD31 in a melanoma tissue microarray. Despite the detection of DEPDC1B expression in all stages of melanoma development, expression levels of SCUBE3 and CD31 were more pronounced and positively correlated in both primary (*n* = 62) and metastatic (*n* = 22) melanomas compared with very low or barely detectable expressions in nevi (*n* = 14) and normal skin tissues (*n* = 2) (**Figure** **7**a,b). As expected, CDC16 was ubiquitously expressed in all the analyzed specimens, but we detected a positive correlation between SOX10 and SCUBE3 expressions in metastatic melanomas (*n* = 19/22) (Figure S9, Supporting Information). Altogether, these results demonstrate that SOX10 and DEPDC1B are clinically correlated with SCUBE3 and CD31 expressions in advanced stages of melanoma, consistent with their regulatory relationship in promoting melanoma angiogenesis and metastasis (Figure 7c).

## 3 Discussion

Human malignant melanoma remains one of the most devastating diseases because of its highly metastatic capability and resistance to current treatments. The ability of melanoma to induce angiogenesis is considered to facilitate metastasis and contribute to the drug resistance. Elucidating the molecular mechanisms that govern melanoma angiogenesis and metastasis will be crucial to understanding disease progression and for developing therapeutic strategies. Here, we demonstrated that SOX10 regulates expression of DEPDC1B which sequesters ubiquitin ligase CDC16 to stabilize the secreted SCUBE3 protein for promoting melanoma growth, angiogenesis, and metastasis (Figure 7c).

Our TCGA analysis revealed that *DEPDC1B* mRNA level was elevated in cutaneous melanoma and was correlated with poor survival. Several previous studies demonstrated that DEPDC1B expression is abnormally upregulated in various cancer types, including pancreatic ductal adenocarcinoma,^[^ ^16^ ^]^ bladder cancer,^[^ ^17^ ^]^ prostate cancer,^[^ ^15^ ^]^ hepatocellular cancer,^[^ ^18^ ^]^ glioblastoma,^[^ ^14^ ^]^ oral cancer,^[^ ^11^ ^]^ and soft‐tissue sarcomas,^[^ ^37^ ^]^ and is associated with poor prognosis, yet the mechanism underlying its dysregulation remains unclear. We found that DEPDC1B expression was positively regulated by an oncogenic driver SOX10, which is required for melanoma formation and progression.^[^ ^27^, ^28^ ^]^ This is based on the finding that DEPDC1B expression was reduced and partly restored in *SOX10 KD* and *SOX10 OE*+*SOX10 KD* cells, respectively. In addition, SOX10 can transactivate the *DEPDC1B* promoter mainly through binding to consensus motifs 2, 1, 3, further indicating that *DEPDC1B* is a direct downstream target of SOX10. A few studies have identified several transcriptional targets of SOX10 that partly contribute to melanoma growth, survival, and metastasis.^[^ ^28^, ^32^, ^38^, ^39^, ^40^ ^]^ Likewise, our epistasis analysis showed the forced expression of DEPDC1B could partially rescue the tumorigenic effects in *SOX10 KD* cells. These results suggest that SOX10 can activate multiple targets, which appear to act in a combinatorial manner to manifest the full‐blown melanoma malignancy, although further studies are required to test this theory. In addition to SOX10, it is possible that other as yet discovered factors could regulate DEPDC1B expression through binding distal regulatory elements (or enhancers). Nevertheless, the regulation of DEPDC1B by SOX10 is further supported by their co‐expression in most biopsies from different stages of melanoma, strongly suggesting that SOX10 is one of the upstream factors contributing to the abnormal upregulation of DEPDC1B expression in melanoma.

Our loss‐of‐function studies using two independent shRNAs demonstrated that DEPDC1B is required for melanoma growth both in vitro and in vivo, which is consistent with previous findings using a single shRNA.^[^ ^20^ ^]^ We further showed that DEPDC1B is crucial for melanoma invasion and metastasis. In contrast to previous studies that found canonical WNT/*β*‐catenin signaling and RHO GTPase signaling function downstream of DEPDC1B to promote cancer cell migration and invasion,^[^ ^10^, ^13^, ^15^ ^]^ we found that *DEPDC1B KD* and *OE* melanoma cells did not exhibit significantly altered signaling activities, ruling out their involvement in mediating the metastatic function of DEPDC1B. However, we observed an increase of microvessel formation in subcutaneous tumors by *DEPDC1B OE*, a marked reduction and increase of microvessel density in the periphery of lung nodules derived from tail vein injection of *DEPDC1B KD* and *OE* cells in mice, respectively. Similar changes in tube formation were observed in HUVECs cultured in CM from *DEPDC1B KD* and *OE* cells. These results suggest the secretion of pro‐angiogenic factors from DEPDC1B‐expressing cells promote melanoma metastasis via induction of angiogenesis. Mass spectrometry analysis of CM from *DEPDC1B KD* and *OE* cells identified SCUBE3, which was both required and sufficient for promoting angiogenesis in vitro and in vivo. In agreement with this, a previous report demonstrated that SCUBE3 mediated angiogenesis in non‐small‐cell lung carcinoma cancer cells,^[^ ^26^ ^]^ although how SCUBE3 expression is regulated was not examined in the study. Our findings showed that DEPDC1B regulated SCUBE3 protein stability rather than its transcription level, as *DEPDC1B KD* and *OE* cells exhibited reduced and prolonged SCUBE3 half‐life, respectively, without dramatically altering mRNA levels. Treatment with proteasome inhibitor MG132 restored SCUBE3 protein level in *DEPDC1B KD* cells, but not with autophagy inhibitor chloroquine, suggesting that SCUBE3 destabilization is mediated through the ubiquitin‐proteasome pathway. Consistently, the level of ubiquitinated SCUBE3 was elevated and reduced in *DEPDC1B KD* and *OE* cells, respectively. In understanding this mechanism, we found the ubiquitin ligase CDC16 is an interacting factor of DEPDC1B in regulating SCUBE3 protein stability as *CDC16 KD* resulted in an increase in SCUBE3 protein level. In addition, CDC16 also interacted with SCUBE3. The stabilization of SCUBE3 in *CDC16 KD* cells could be mimicked by the preferential association of CDC16 with excess amounts of DEPDC1B protein, thereby preventing SCUBE3 from binding to CDC16 for degradation. Recent studies showed that DEPDC1B induces ERK protein phosphorylation to enhance N‐Myc protein stability in neuroblastoma cell.^[^ ^41^ ^]^ It is therefore possible that DEPDC1B adopts distinct molecular mechanisms to regulate protein stability in different cancer types, underlying its context‐dependent functions. In contrast, other studies showed that Dishevelled (DVL), a positive regulator of Wnt signaling, is required for ZNRF3/RNF43‐mediated ubiquitination and degradation of Wnt coreceptor Frizzled (FZD). The downregulation of FZD is mediated through the binding of FZD to the DEP domain of DVL,^[^ ^42^ ^]^ raising the possibility that the DEP domain of DEPDC1B could mediate protein destabilization, but this remains to be determined.

Our findings demonstrated that *SCUBE3 OE* partially restored melanoma angiogenesis and metastasis in *DEPDC1B KD* cells, implying additional factors are required. Indeed, beside SCUBE3, the mass spectrometry analysis also revealed several secreted factors were regulated by DEPDC1B at the transcriptional and possibly post‐translational levels. In addition, CM from non‐SCUBE3‐expressing and less aggressive primary melanoma SK‐MEL‐28 cells treated with *DEPDC1B KD* and *OE* showed a negative and positive effect on HUVEC tube formation, respectively, suggesting that other pro‐angiogenic factors regulated by DEPDC1B could contribute to primary melanoma angiogenesis and progression. This is in line with a relatively low level of SCUBE3 expression in DEPDC1B^+^ primary tumor samples. How these secreted factors are regulated by DEPDC1B and their contribution to its oncogenic properties at different stages of melanoma development warrant further investigation.

In addition, immunofluorescence staining in melanoma tissue arrays showed that expression levels of SOX10, DEPDC1B and CDC16 were high in nevi which do not metastasize but SCUBE3 was barely detectable. These results underlie distinct transcriptional regulatory mechanisms at different stages of melanoma development in which SCUBE3 is transcriptionally silenced in nevi and expression becomes activated and stabilized at the protein level by DEPDC1B in primary and metastatic melanomas respectively. Alternatively, DEPDC1B might lose its ability to sequester CDC16 in nevi, resulting in SCUBE3 destabilization even if it is expressed. A recent report showed that DEPDC1B regulated the progression of human chordoma through UBE2T‐mediated ubiquitination of BIRC5.^[^ ^43^ ^]^ It is therefore possible that DEPDC1B could destabilize SCUBE3 in nevi through association with unknown factors to mediate ubiquitination. Further investigations are needed to clarify these issues.

## 4 Conclusion

This study unravels a new molecular mechanism of DEPDC1B acting downstream of SOX10 to promote melanoma angiogenesis and metastasis through CDC16 sequestration that stabilizes secreted SCUBE3, which suggests the possibility of targeting SCUBE3 as a therapeutic strategy against metastatic melanoma.

## 5 Experimental Section

### TCGA Database Mining

The expression profiles of *DEPDC1B* in melanoma patients compared with normal samples, and the survival curve grouped by different *DEPDC1B* expression levels were generated from GEPIA (http://gepia2.cancer‐pku.cn/#index). The copy number variation of *DEPDC1B* was obtained from cBioportal for cancer genomes (https://www.cbioportal.org/) under the category of melanoma.

### Cell Culture and Plasmids

Human melanoma cell lines WM266‐4, A2058, and SK‐MEL‐28 were purchased from American Type Culture Collection (ATCC), and HUVECs were a kind gift from Dr. Annie Cheung. Purchased cell lines were tested to exclude mycoplasma contamination by the Centre for PanorOmic Sciences, Li Ka Shing Faculty of Medicine, HKU. Both WM266‐4 and SK‐MEL‐28 cells were cultured in 10% serum‐containing EMEM (Sigma), A2058 and human embryonic kidney 293T cells were cultured in 10% serum‐containing DMEM (Gibco), and HUVECs were cultured in Medium 200RPF (Gibco) with low serum growth supplement (ThermoFisher) according to the manufacturer's instruction. All cell lines were maintained in a 37 °C humidified incubator supplemented with 5% CO_2_. The list of culture conditions for each cell line is shown in Table S5 (Supporting Information).

Plasmids encoding V5‐DEPDC1B, SOX10, SCUBE3, CDC16‐HA, and truncated CDC16 N1‐266‐HA and N262‐620‐HA were used. These genes were amplified from WM266‐4 cDNAs and cloned into pLVX‐IRES‐EF1a‐puro vector using restriction enzymes. The primer sequences and enzyme sites are listed in Table S6 (Supporting Information). A pLKO.1‐TRC vector was used to knockdown *DEPDC1B*, *SOX10*, *SCUBE3*, and *CDC16*. The shRNAs were generated by IDT and cloned into pLKO.1‐TRC at the AgeI and EcoR1 sites. The shRNA target sequences are listed in Table S7 (Supporting Information).

### Materials and Reagents

qPCR primers and antibodies used in the study are listed in Tables S8 and S9 (Supporting Information). Rabbit anti‐DEPDC1B antibody was custom‐made by GenScript and raised against antigen sequence: CISPEEFEYQRSYGS. Protein synthesis inhibitor cycloheximide (10 mg mL^–1^) was purchased from Santa Cruz (sc‐3508), proteasome inhibitor MG132 (5 µm) from SelleckChem (S2619), recombinant SCUBE3 protein from R&D (7730‐SC), and autophagy inhibitor Chloroquine (100 µm, Sigma) was provided by Dr. Stephanie Ma.

### AlamarBlue Assay

Melanoma cell proliferation rate was measured using alamarBlue cell viability reagent (Invitrogen). Melanoma cells were seeded in a 96‐well plate at a density of 1000 cells per well for A2058, 1500 cells per well for WM266‐4, and 2000 cells per well for SK‐MEL‐28. The proliferation rate was assayed by incubating cells in tenfold diluted alamarBlue reagent at 37 °C for 2 h. The absorbance was measured at 570 nm and 600 nm using a multi‐plate reader and the proliferation rate was calculated according to the manufacturer's instructions. The proliferation assay was monitored every day or every other day according to the growth rate and was stopped when the growth rate reached 100%.

### EdU Staining Assay

Melanoma cell proliferation ability was measured by EdU Click‐it kit (ThermoFisher). Melanoma cells were seeded onto coverslips in a 24‐well plate to ensure the confluency was less than 90% at the end of the assay. Half of the medium was replaced with fresh medium containing EdU at a final concentration of 10 × 10^−6^ m. The WM266‐4 and SK‐MEL‐28 cells were incubated with EdU for 16 h and A2058 cells were incubated for 4 h. Cells were fixed, washed, and permeabilized before staining. The Click‐it reaction cocktail was prepared according to the manufacturer's instructions and incubated with the cells protected from light. DAPI (1 µg mL^–1^) was used to counterstain the nuclei before imaging under an inverted fluorescence microscope (Olympus IX71 with Olympus DP71 color digital camera). Nuclei and EdU positive cell numbers were measured by Image J software.

### Colony Formation Assay (CFA)

Cells transduced with KD or OE lentivirus were digested, resuspended as single cells, and seeded in a 6‐well plate at 2500 cells per well for WM266‐4, 1500 cells per well for A2058, and 3000 cells per well for SK‐MEL‐28. Half of the culture medium was refreshed daily to maintain proper cell growth. After 2 to 3 weeks, crystal violet staining was performed to determine colony formation status. Images were captured and the numbers of colonies were counted by Image J.

### Transwell Invasion Assay

For the 2D transwell invasion assay, melanoma cells with different treatments were cultured for 3 d before the assay. The upper chamber of transwell insert (Corning, #353097) was coated with Matrigel (BD, 1 mg mL^–1^) at 37 °C for 4 h. Cells were digested and resuspended as single cells in serum‐free culture medium. The cells were seeded in the upper chamber of the transwell in a 24‐well plate with 500 µL serum‐containing (10%) culture medium at 100 000 cells per well for WM266‐4 and incubated for 24 h, 50 000 cells per well for A2058 for 16 h, and 50 000 cells per well for SK‐MEL‐28 for 24 h. Cells on the transparent membrane were then washed, fixed, and counterstained with DAPI. Images were obtained under a 10X lens using an inverted fluorescence microscope (Olympus IX71 with Olympus DP71 color digital camera) for eight to ten random views. Cell numbers were counted by Image J.

### 3D Spheroid Invasion Assay

Melanoma cells subjected to different treatments for 24 h were digested and resuspended as single cells. Low‐melting point agarose (2% w/v) in PBS solution was prepared by heating to 65 °C, and then cast into a 96‐well plate and allowed to cool down at room temperature. Melanoma spheroids were prepared using the liquid overlay method. Briefly, 100 µL of melanoma cells (50 000 mL^–1^) were added onto a 96‐well plate coated with agarose gel and incubated for 72 h to allow the formation of three‐dimensional spheroids. Rat tail collagen I (BD, #354236) at tenfold dilution was prepared and 50 µL was layered onto a 96‐well plate and incubated at 37 °C for 30 min for gel solidification. Spheroids were transferred by a P200 pipette onto the collagen gel and embedded by overlaying an additional 100 µL of collagen. After 30 min incubation, the culture medium was overlaid on top of the solidified collagen. Images of spheriods on the day of embedding and after 72 h of incubation were taken using an Olympus CKX41 inverted microscope. The spheroid invasion area was calculated using Image J.

### Conditioned Medium Collection and Concentration

Cells with different treatments were seeded one day before harvesting to reach 80% confluency. After 24 h, cells were rinsed three times using serum‐free medium and then cultured with the same volume of serum‐free medium for 48 h. The medium was collected and filtered with a 0.45 µm filter and stored at −80 °C until use. For Western blotting, the conditioned medium was concentrated by a centrifugal device (Nanosep) according to the manufacturer's instructions.

### HUVEC Tube Formation Assay

The HUVECs were cultured in phenol red‐free M200 medium (ThermoFisher) with low serum growth supplement (Gibco). A 24‐well plate was coated with 100 µL reduced growth factor basement membrane matrix (Gibco, 50 µL per cm^2^) per well and incubated at 37 °C for 30 min to allow gel solidification. The HUVECs were harvested and diluted in nonsupplemented medium at a density of 4.2 × 10^4^ cells per 200 µL and then mixed with 200 µL conditioned medium. The mixture was gently added to each well and incubated at 37 °C. Tube formation was monitored every 2 h and images were taken using an Olympus CKX41 inverted microscope. Tube length was quantified by Image J software using the Angiogenesis Analyzer plugin.

### In Vivo Xenograft Assay

All animal studies were carried out under the research protocol CULATR 4265‐17 and 5356‐20 approved by the Committee of the Use of Live Animals in Teaching and Research (CULATR) at the University of Hong Kong. All animal work and procedures were strictly followed according to the Animals (Control of Experiments) Ordinance (Hong Kong) and the Institute's guidance from Centre for Comparative Medical Research (CCMR), Li Ka Shing Faculty of Medicine, The University of Hong Kong. BALB/cAnN‐nu mice were used in xenograft experiments. All mice were provided by and housed in specific pathogen free area in the CCMR.

The WM266‐4 cells with the indicated treatments were cultured, digested, and resuspended as single cells. The cells were washed twice using serum‐free EMEM with centrifugation and resuspension. The cell concentration was diluted with Matrigel at a 4∶1 ratio to a final density of one million cells per 100 µL. For each immunodeficient mouse, 100 µL of the cell mixture was injected subcutaneously into the right flank. Tumor formation was monitored and imaged. The tumors were dissected at the end of the experiment and weighed. The excised tumors were sectioned and subjected to histological and immunostaining analysis.

### In Vivo Lung Metastasis assay

Melanoma cells were labeled with a promoter driven firefly luciferase reporter together with the desired gene treatments. Cells were digested, washed twice with serum‐free culture medium, and resuspended at a density of one million cells per 100 µL. For each mouse, 100 µL of the cell mixture was injected via the tail vein of NOD/SCID mice. At 7 weeks post‐injection, mice were injected with D‐luciferin intraperitoneally and then subjected to bioluminescence imaging. At the end of the experiment, mice were sacrificed by anesthetic overdose and lung tissues were collected for histological analysis.

### Matrigel Plug Assay

For in vivo evaluation of angiogenesis, Matrigel plug assay was performed in 6‐week‐old C57BL/B6 mice. Mice were anesthetized and injected subcutaneously in both flanks with 0.3 mL ice‐cold Matrigel (10 mg mL^–1^) mixed with heparin (64 U mL^–1^) alone as control group, or together with recombinant SCUBE3 protein (600 ng mL^–1^). When CM was applied, 15 mL CM was collected from the indicated treatment and further concentrated to 200 µL which was then mixed with 2 mL heparin containing Matrigel solution before subcutaneous injection into C57BL/6 mice. Within days, cells from the surrounding tissues migrated into the Matrigel to form vascular network. At 7 d post‐injection, mice were sacrificed to excise the Matrigel plug to assess the angiogenic response. The formation of neo‐vessels was indirectly determined by measuring the hemoglobin content using the Drabkin's reagent kit according to the manufacturer's protocol. Histological analysis of fixed and paraffin‐embedded Matrigel plugs was performed using H&E staining and antibody immunofluorescent staining.

### Chromatin Immunoprecipitation and Quantitative Polymerase Chain Reaction (CHIP‐qPCR)

CHIP‐qPCR assay was performed to examine the protein‐DNA interaction using a Pierce magnetic CHIP kit (ThermoFisher). For each reaction, two million melanoma cells were cross‐linked by formaldehyde (final concentration 1%) at room temperature for 10 min and quenched by glycine at room temperature for 5 min. The cells were then washed and scraped into 1 mL PBS supplemented with proteinase inhibitors, and then PBS was removed by centrifugation. Nuclei were isolated by lysing with membrane extraction buffer followed by centrifugation. Diluted micrococcal nuclease (MNase; 1∶10, 2.4 µL) was added to the nuclei and resuspended in 200 µL MNase digestion buffer and incubated at 37 °C for 15 min with inversion every 5 min. The digestion was stopped by adding 20 µL stop solution followed by centrifugation. The nuclei were recovered in 100 µL IP dilution buffer and sonicated using a BioruptorPico sonication system (Diagenode) at 4 °C for 30 s with a 30‐s pulse. Three cycles were used for WM266‐4 cells and four cycles were used for A2058 cells. After sonication and centrifugation, 10 µL of the supernatant was kept as the input and the remaining 90 µL was used for immunoprecipitation with 5 µg anti‐SOX10 antibody (ThermoFisher, #PA5‐40697) or normal rabbit IgG control overnight at 4 °C. The following day, the protein‐DNA complex was isolated using 20 µL magnetic beads. The purified DNA served as the templates for the qPCR analysis using primers flanking the binding motifs on the *DEPDC1B* promoter region. The ChIP‐qPCR primers used are listed in Table S10 (Supporting Information).

### Co‐Immunoprecipitation (IP) and Mass Spectrometry (MS)

Cells were lysed in IP lysis buffer and centrifuged to obtain cell lysate in the supernatant. The protein concentration was then determined using a BCA assay kit (ThermoFisher). For each reaction, 2 mg protein lysate was mixed with a specific antibody or normal IgG control in a ratio of 1000∶1 and incubated overnight at 4 °C to allow the immunocomplex to form. Pierce MS‐compatible magnetic IP kit (ThermoFisher) was used to perform the pull‐down. The beads were mixed homogenously with gentle vortexing and 25 µL was used in each reaction after two washes in IP lysis buffer. The antigen‐antibody complex was incubated with the pre‐washed beads at 4 °C for 3 h. A magnetic stand was used to collect the beads. The beads were washed three times in wash buffer A and another two times in wash buffer B, and the proteins bound to the beads were then eluted using 2x Laemmli loading buffer supplemented with 10% *β*‐ME at 95 °C for 10 min. Proteins were subjected to SDS‐PAGE and transferred to PVDF membrane for the immunoblotting analysis. The detailed Western blotting procedures are described in the supporting information. For the mass spectrometry analysis, proteins were stacked at the border between the stacking gel and the separating gel and the gel slices were excised and submitted to HKU proteomics and metabolomics core.

### Tissue Immunofluorescence Staining (IF)

Mouse lungs were washed, fixed in neutral buffered formalin, and then dehydrated in increasing concentrations of ethanol. At the end of the dehydration, the ethanol was replaced with xylene and then by paraffin wax. The air was removed in a 60 °C vacuum oven for 1 h before embedding. Sectioning was performed to obtain 5 µm thick slides. For immunofluorescence staining, the wax was melted in a 65 °C oven for 15 min and slides were processed sequentially in the following order: 100% xylene 10 min twice, 100% ethanol 3 min twice, 95% ethanol 2 min twice, 70% ethanol once, and distilled water twice. Antigen retrieval was performed by boiling the slides in target retrieval solution citrate pH 6 (Dako) for 10 min. After cooling to room temperature, slides were washed in TBST (1x TBS with 0.1% Triton‐X) and blocked in 1% NDS diluted in TBST for 1 h at room temperature and then incubated with the primary antibody overnight at 4 °C. The primary antibodies were removed and washed using TBST for 5 min four times. Secondary antibodies conjugated with a fluorophore were diluted in 1% NDS and incubated with the slides at room temperature for 1 h protected from light. Cell nuclei were counterstained with DAPI (1 µg µL^–1^). The slides were washed with TBST four times prior to mounting and imaging.

### Melanoma Tumor Tissue Microarray (TMA)

Human paraffin‐embedded melanoma tissue arrays (#ME1004h) were purchased from US Biomax Inc. The processing procedures were the same as mentioned above. Images were captured using LSM 780 confocal system (Carl Zeiss) maintained by the Faculty Core Facility, Li Ka Shing Faculty of Medicine, HKU and analyzed by ZEN blue software (Carl Zeiss).

### Statistical Analysis

The statistical analysis of all data was performed by GraphPad Prism 9. All data were presented as mean ± SD from at least three independent experiments except the bioluminescence data were present from minimal to maximum and the scores of SCUBE3/CD31 were present as median with individual values. Unpaired Student′s t‐test and ordinary one‐way ANOVA were used in comparison between two groups and more than two groups in column analysis separately. Two‐way ANOVA was used in comparison of group analysis. Simple linear regression was used to analyze the correlation between % of tumor area and % of blood vessel area. The statistical analyses performed by significant differences were evaluated by *p* value. A *p* value less than or equal to 0.05 was considered statistically significant. A *p* value less than 0.05 is marked by *, *p* value less than 0.01 is marked by **, *p* value less than 0.001 is marked by ***, and *p* value less than 0.0001 is marked by ****.

## Conflict of Interest

The authors declare no conflict of interest.

## Author Contributions

Conceptualization: F.H., X.T.Y., and M.C.; methodology: F.H., K.O.F., M.P.L.C., J.A.I.L., R.L., T.W.L., R.S., X.T.Y., and M.C.; investigation: F.H., K.O.F., R.L., T.W.L., and X.T.Y.; resources: F.H., K.O.F., M.P.L.C., J.A.I.L., R.L., T.W.L., P.P‐C. I., and X.T.Y.; writing–original draft: F.H. and M.C.; writing–review & editing: F.H. and M.C.; project supervision: M.C. and X.T.Y.; funding acquisition: J.A.I.L., R.L., X.T.Y., and M.C.

## Supporting information

Supporting Information

Supplemental Table 1

Supplemental Table 2

Supplemental Table 3

Supplemental Table 4

## Data Availability

The data that support the findings of this study are available in the supplementary material of this article.
