# Supplementary material for: DEPDC1B Promotes Melanoma Angiogenesis and Metastasis through Sequestration of Ubiquitin Ligase CDC16 to Stabilize Secreted SCUBE3
Source: Adv Sci (Weinh). 2022 Jan 27;9(10):2105226. doi: 10.1002/advs.202105226 (PMC8981904; doi:10.1002/advs.202105226)
Supplement: Supplementary file 1 — Supporting Information [file ADVS-9-2105226-s003.pdf]

ADVANCED SCIENCE
Open Access

Supporting Information

for *Adv. Sci.*, DOI: 10.1002/advs.202105226

DEPDC1B promotes melanoma angiogenesis and metastasis through sequestration of ubiquitin ligase CDC16 to stabilize secreted SCUBE3

*Feng Hu, Ki On Fong, May Pui Lai Cheung, Jessica Aijia Liu, Rui Liang, Tsz Wai Li, Rakesh Sharma, Philip Pun-Ching IP, Xintao Yang, Martin Cheung**

Supporting information

DEPDC1B promotes melanoma angiogenesis and metastasis through sequestration of ubiquitin ligase CDC16 to stabilize secreted SCUBE3

*Feng Hu, Ki On Fong, May Pui Lai Cheung, Jessica Aijia Liu, Rui Liang, Tsz Wai Li, Rakesh Sharma, Philip Pun-Ching IP, Xintao Yang, Martin Cheung**

Supporting Experimental Section

Third generation lentivirus package and infection

293T cells were seeded in 100mm-dish and cultured to reach 95-100% confluency. The culture medium was refreshed with 9 mL fresh medium 1 hour prior to virus packaging. Using PolyJet transfection reagent (SignaGen), 7.5 µg target plasmid together with 1.875 µg envelop plasmid pMD2.G and 5.625 µg packaging plasmid psPAX2 were transfected into 293T cells. In details, 40 µL Polyjet and total 15 µg DNA were diluted in 500 µL serum-free DMEM separately and vortex to make homogenous mix. Add the diluted Polyjet reagent to the DNA mix immediately all at once followed by a 10-minute incubation at room temperature. Then the Polyjet-DNA mix was added dropwise to 293T cell medium and mixed gently by swirling the dish. 24 hours post transfection, the culture medium was refreshed, and the supernatant were harvested 48 hours and 72 hours post transfection. Altogether, the lentivirus-containing supernatant was filtrated through 0.22 µm filter (Millipore), aliquoted into 1 mL Eppendorf tubes and store in -80°C deep fridge.

Polybrene (8 µg μL^{-1} stock, Santa Cruz) was used at a 1:2000 ratio for lentivirus infection. Cells were seeded in 6-well plate or dish to 30-40% confluency before infection. Polybrene was added in each well followed by 10-15 minutes' incubation. Proper volume of lentivirus-containing supernatant was added to the cells. Selection markers like puromycin (Life Technologies, 1 µg μL^{-1} stock) was diluted at 1:2000 scale in infected cell for 48 hours to select stable cell lines. 3-4 days post infection, cells were collected for the following stated assays.

RNA extraction and real time qPCR

Cells were lysed and extracted for total RNA according to the manufacturer's instruction using TaKaRa MiniBEST universal RNA extraction kit (TaKaRa). In details, four days post gene regulation, cells were lysed in buffer RL supplemented with DTT, and incubate at room temperature for two minutes. All the lysates were transferred to gDNA eraser spin column in a collection tube followed by 1-minite centrifuge at 12000 rpm. The flow-through was mixed thoroughly with equal volume of 70% ethanol and applied to RNA spin column in a collection tube followed by 1-minute centrifuge at 12000 rpm. The RNA column was then successively washed by buffer RWA and buffer RWB. In order to remove any possible DNA residues, recombinant DNase was applied on the silica membrane of the column for a room-temperature digestion for 15 minutes. The column was then washed twice with buffer RWB and centrifuged for 2 more minutes to remove any ethanol residue. To elute the RNA, RNase-free water was added directly on the membrane and incubate for 5 minutes at room temperature. Centrifuge at 12000 rpm for 2 minutes to elute and measure RNA concentration with NanoDrop 2000 (ThermoFisher).

PrimeScript RT reagent kit (TaKaRa) was used to generate cDNA for quantitative real-time PCR analysis or serve as PCR template according to the manufacturer's protocol. To analyze the expression level of a target gene, real-time qPCR was applied using ChamQ SYBR color qPCR master mix (Vazyme). The reaction solution was prepared in a hard-shell 96-well qPCR plate (Bio-Rad).

Western Blot and Immuno-blotting

Cells infected with desired gene treatment were lysed with RIPA lysis buffer (150mM NaCl, 1mM EDTA, 1% NP40, 0.5% Sodium deoxycholate, 0.1% SDS, 50mM Tris-HCl, pH 7.5) or IP lysis buffer (150 mM NaCl, 1 mM EDTA, 1% NP-40, 25 mM Tris-HCl, 5% Glycerol, pH

7.4) depending on the assay and the formulars are shown as below table. Protease and phosphatase inhibitors cocktail (100x, ThermoFisher, #78430) was supplemented to the lysis buffer before lysing cells. For thorough lysis, the cell lysate was incubated on ice for 40 minutes with intermittent vortex every 10 minutes. After protein concentration measurement, 40 – 60 μg of proteins lysates were mixed with 4x Laemmli loading buffer supplemented with 10% β-mercaptoethanol (β-ME) (Bio-Rad, #1610747) and heated at 95°C for 5 minutes to denature the proteins. Proteins were stacked and separated through SDS-PAGE and subjected to antibody incubation. ECL HRP substrate (advansta, #K-12045-D50) was to give out chemiluminescence signals. Film-image the signal using darkroom development techniques. The signal intensity was controlled by exposure time. After proper fixation, the films were washed in running water, oven-dried and scanned to keep the image. The protein bands were quantified using Image J.

RHOA/RAC1 activation assay

The RHOA activation assay was done using Rhotekin RBD beads (Cytoskeleton, #RT02). The RAC1 activation assay was done using PAK-PBD beads (Cytoskeleton, #PAK02). Melanoma cells with desired treatment were cultured no more than 70% confluency. Cells were washed using ice-cold PBS and collected in IP lysis buffer supplemented with 1% proteinase inhibitors. Followed by 5-minute lysis on ice, the cell debris was separated using centrifugation at 16,000rcf for 3 minutes. Supernatant containing the active RHOA/RAC1 was transferred to a new 1.5 mL tube and 50 μL were saved for protein concentration measurement. 300-500 μg total protein was incubated with either Rhotekin beads (50 μg per reaction) or PAK-PBD beads (10 μg per reaction) at 4°C for 2 hours. Beads were then collected by centrifugation at 4°C at a speed of 1,000 rcf for 3 minutes and washed for 4 times. Proteins bound to the beads were then eluted using 2x Laemmli buffer and denatured under 95°C for 10 minutes. All volume obtained was loaded into 12% SDS-PAGE for further western blot analysis.

β-catenin TOPFlash luciferase reporter assays

To test the activity of β-catenin, 2 μg DNA in total containing M50 8x TOPFlash and Renilla in a ratio of 10:1 was transfected into 106 melanoma cells using polyjet. The negative control was transfected with only Renilla without the presence of any reporter. Cells were collected 48 hours after transfection and lysed in passive lysis buffer provided in the Dual-luciferase Reporter Assay System kit (Promega, #E1910) on a shaker at room temperature for 15 minutes. 20 μL cell lysate was added each well in an untransparent 96-well plate followed by addition of 100 μL LAR II solution and subjected to firefly luciferase measurement. 100 μL stop solution was added afterwards for the Renilla luciferase measurement. One empty well containing no cell lysate was also measured for background signal. Luciferase activity relative to Renilla was calculated and normalized with control group.

Calculation method using Image J

The calculation of "blood vessel total area" on xenograft tissue sections in Figure 2g was performed based on the immunofluorescent staining against CD31. Two xenograft tumors from each group were sectioned and three adjacent sections were picked for H&E staining, fluorescent-IHC staining and DAB-IHC staining respectively. Triplicates were obtained every 100 μm apart and five random images were taken in each section subjected to calculation by Image J software. $n = 2 \times 3 \times 5 = 30$. The calculation of "blood vessel total area" and "tumor total area" on lung metastasis tissue sections in Figure 2h were performed based on the H&E staining. 7 lungs dissected from intravenous injection were fixed and sectioned for H&E staining. High magnification images were taken and stitched into a full section image before subjected to analysis using Image J. Blood vessel area containing red blood cell and tumor nodule area darkly stained were identified by color threshold identification function and the area was measured.

Supporting Figures

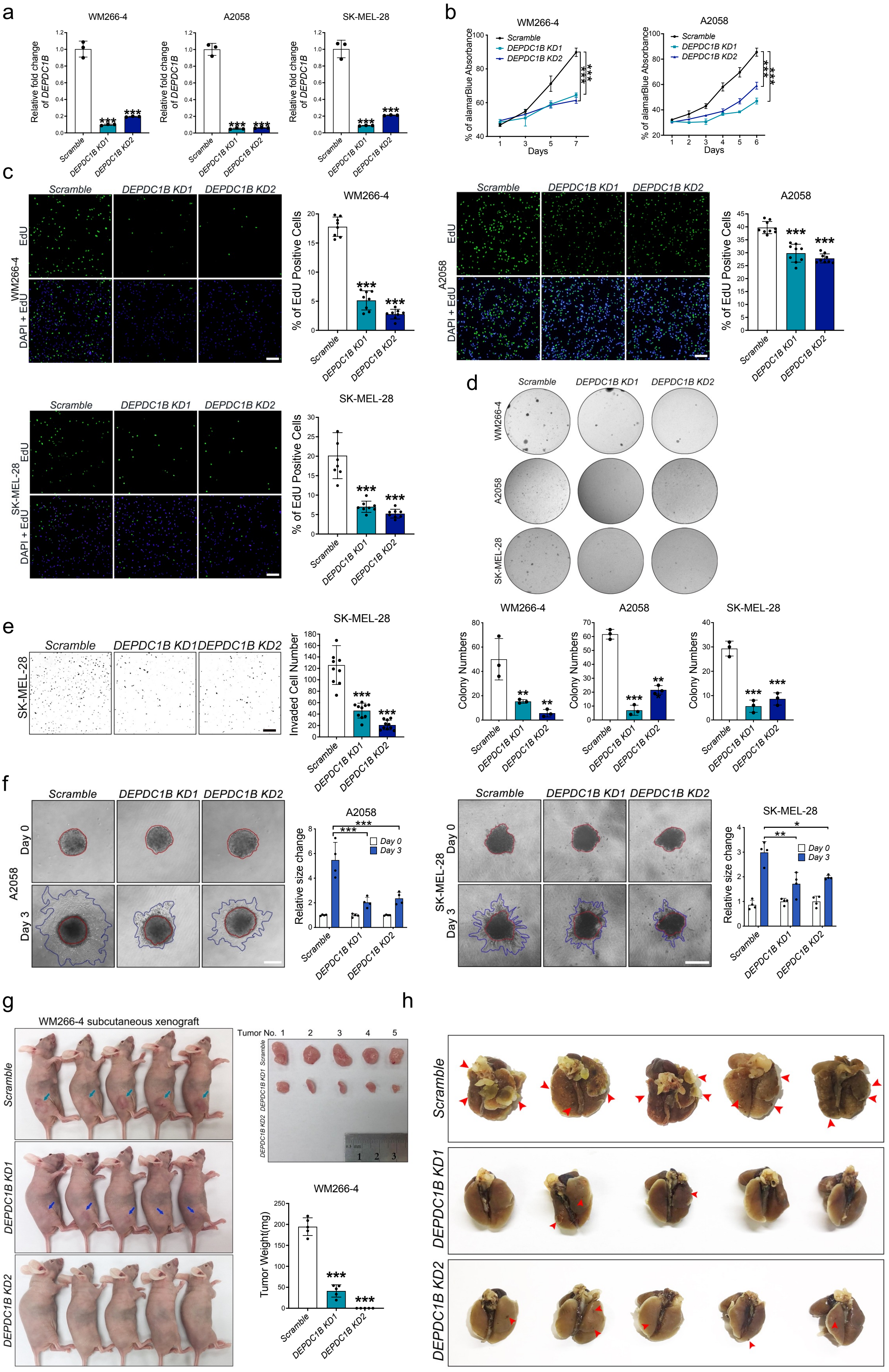

Figure S1. Reduced *DEPDC1B* expression attenuates melanoma growth, tumorigenicity, invasion and lung metastasis. a) qPCR analysis of *DEPDC1B* mRNA levels in WM266-4, A2058 and SK-MEL-28 cells transduced with control (*scramble*), *DEPDC1B KD* (*DEPDC1B KD1* and *DEPDC1B KD2*), $n = 3$. b) AlamarBlue assay of WM266-4 and A2058 cells treated with the indicated constructs, $n = 4$. c) Representative images of EdU staining and quantification of EdU^{+} WM266-4, A2058 and SK-MEL-28 cells treated with *DEPDC1B KDs*, $n \geq 7$. Scale bar = 200 μm. d) Representative images of WM266-4, A2058 and SK-MEL-28 cells subjected to colony formation assay and quantification of colony number, $n = 3$. e) Representative images of 2D transwell-invaded SK-MEL-28 cells treated with *DEPDC1B KDs*. $n \geq 7$. Cell number was counted using ImageJ software. Scale bar = 200 μm. f) Representative images of 3D A2058 and SK-MEL-28 spheroids invasion assay. $n = 4$. Scale bar = 100 μm. Red dotted lines mark the margin of spheroids on the embedding day (Day 0) and blue dotted lines mark the margin of the invaded spheroids 3 days after embedding (Day 3). Pixel analysis of images was performed to calculate the total area occupied by the tumor spheroids. $n = 4$. Scale bar = 100μm. g-h) Gross images of the subcutaneous xenografts, dissected tumors and metastasis lungs derived from WM266-4 cells with *DEPDC1B KDs*. Green arrows indicate the tumor position of the *scramble* group, while blue arrows indicate the tumor position of the *DEPDC1B KD1* group. Red arrowheads indicate lung nodules. Data are mean ± SD. $^{*}P<0.05$; $^{**}P<0.01$; $^{***}P<0.001$ from from ordinary one-way ANOVA and two-way ANOVA.

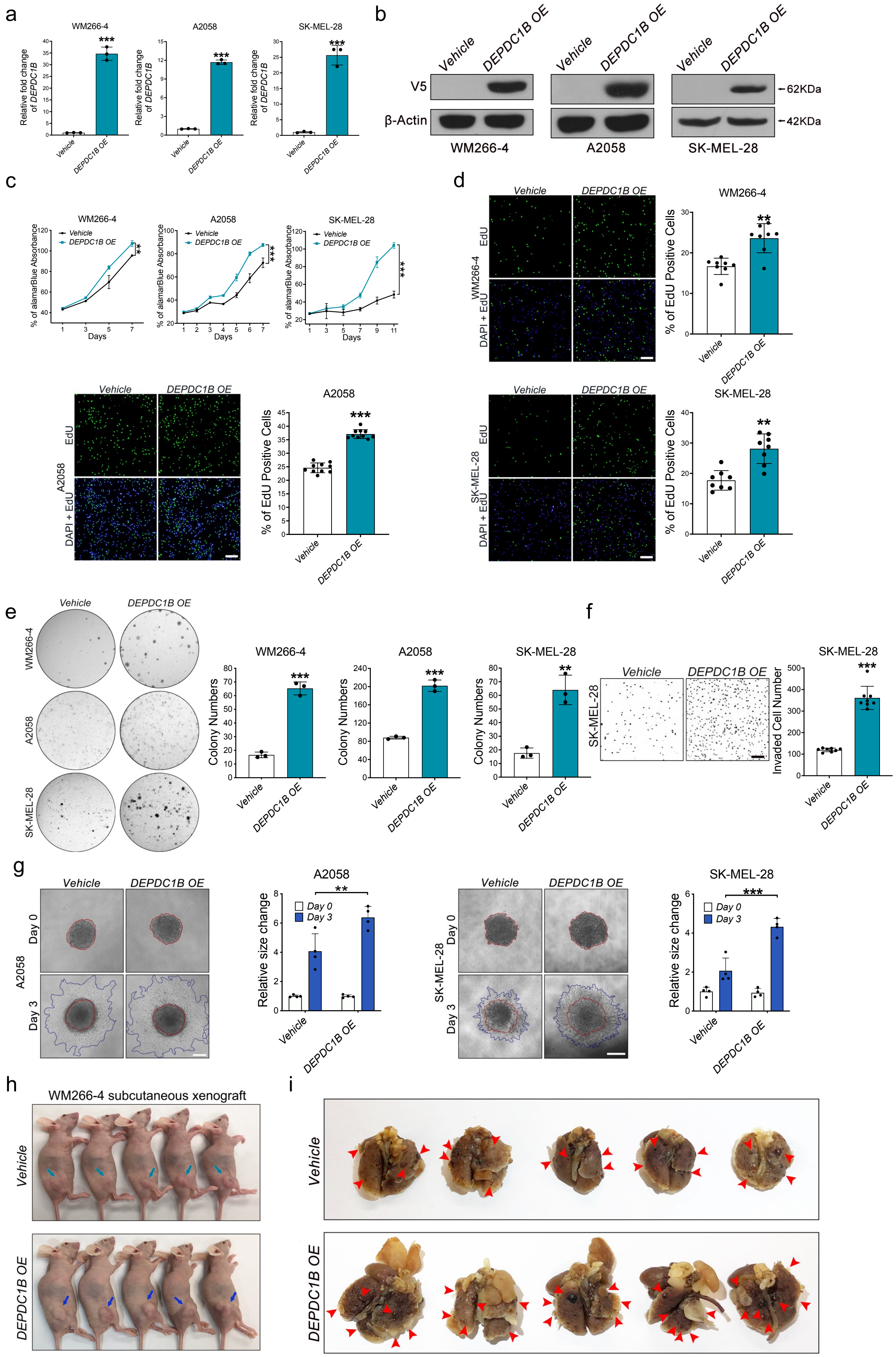
a
WM266-4
A2058
SK-MEL-28
Relative fold change of DEPDC1B
Vehicle
DEPDC1B OE
b
V5
β-Actin
62KDa
42KDa
c
% of alamarBlue Absorbance
Days
d
EdU
DAPI + EdU
% of EdU Positive Cells
e
Colony Numbers
f
Invaded Cell Number
g
Day 0
Day 3
Relative size change
h
WM266-4 subcutaneous xenograft
i

Figure S2. Overexpression of DEPDC1B promotes melanoma growth, tumorigenicity, invasion and lung metastasis.

a) qPCR (n = 3) and Western blot analysis (b) for mRNA and protein levels of control (*vehicle*) and *DEPDC1B* overexpression (*DEPDC1B OE*) in WM266-4, A2058 and SK-MEL-28 cells. c) AlamarBlue of cells treated with *DEPDC1B OE*, n = 4. d) Representative images of EdU staining and quantification of EdU^+ melanoma cells treated with *DEPDC1B OE.* $n \geq 8$. Scale bar = 200 μm. e) Colony formation assay of melanoma cells treated with the indicated constructs and quantification of colony number, n = 3. f) Representative images of transwell-invaded SK-MEL-28 cells, $n \geq 7$, scale bar = 200 μm. g) Representative images of A2058 and SK-MEL-28 3D spheroids invasion assay with *DEPDC1B OE*. Scale bar = 100 μm. n = 4. Red dotted lines mark the margin of the spheroid on the embedding day (Day 0), blue dotted lines mark the margin of the invaded spheroids 3 days after embedding (Day 3). h-i) Gross image of the subcutaneous xenografts and metastasis lungs derived from WM266-4 cells treated with *DEPDC1B OE*. Green arrows indicate the tumor position of the *vehicle* group, while blue arrows indicate the tumor position of the *DEPDC1B OE* group. Red arrowheads indicate lung nodules. Data are mean ± SD. *P<0.05; **P<0.01; ***P<0.001 from unpaired Student′s t-test and two-way ANOVA.

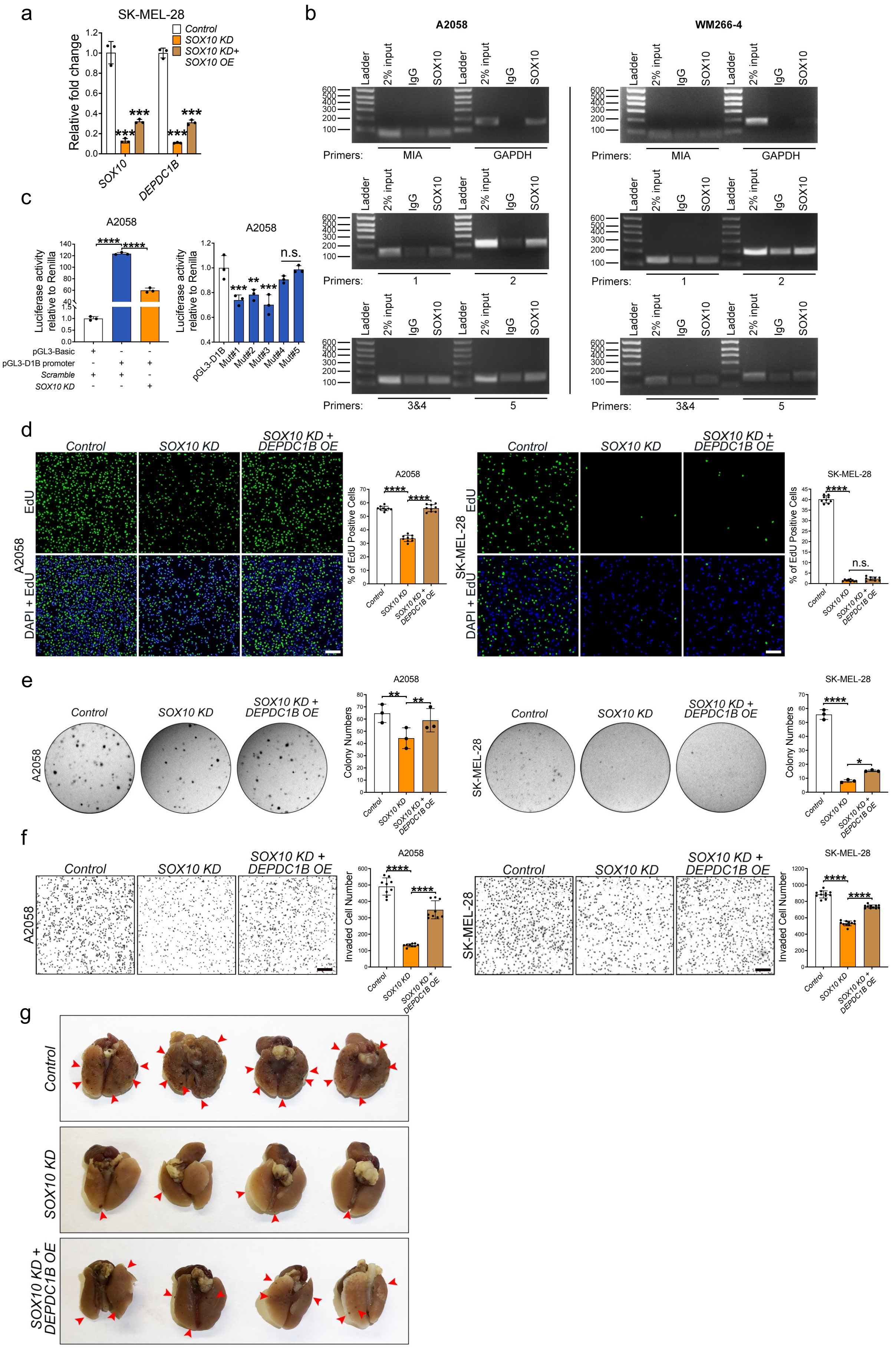
a
SK-MEL-28
Relative fold change
Control
SOX10 KD
SOX10 KD+ SOX10 OE
SOX10
DEPDC1B
b
A2058
WM266-4
Ladder
2% input
IgG
SOX10
Primers:
MIA
GAPDH
1
2
3&4
5
c
A2058
Luciferase activity relative to Renilla
pGL3-Basic
pGL3-D1B promoter
Scramble
SOX10 KD
pGL3-D1B
Mut#1
Mut#2
Mut#3
Mut#4
Mut#5
n.s.
d
Control
SOX10 KD
SOX10 KD + DEPDC1B OE
EdU
DAPI + EdU
A2058
SK-MEL-28
% of EdU Positive Cells
e
Colony Numbers
f
Invaded Cell Number
g
Control
SOX10 KD
SOX10 KD + DEPDC1B OE

Figure S3. SOX10 regulates DEPDC1B expression in melanoma cells. a) qPCR analysis of *SOX10* and *DEPDC1B* mRNA levels in SK-MEL-28 cells treated with the indicated constructs. $n = 3$. b) DNA gel electrophoresis of the products from the ChIP-qPCR analysis. c) Full length *DEPDC1B* promoter activity and the mutated promoter activities were determined compared with the wild-type promoter in A2058 cells. d-f) Representative images and quantification of EdU^{+} melanoma cells, colony formation ability and transwell-invaded A2058 and SK-MEL-28 cells treated with *SOX10 KD* and *DEPDC1B OE* rescue. Scale bar = 200 µm. g) Gross images of the mouse lungs derived from the indicated treatment groups. Red arrowheads indicate lung nodules. Data are mean ± SD. n.s. when $P>0.05$; *$P<0.05$; **$P<0.01$; ***$P<0.001$; ****$P<0.0001$ from ordinary one-way ANOVA and two-way ANOVA.

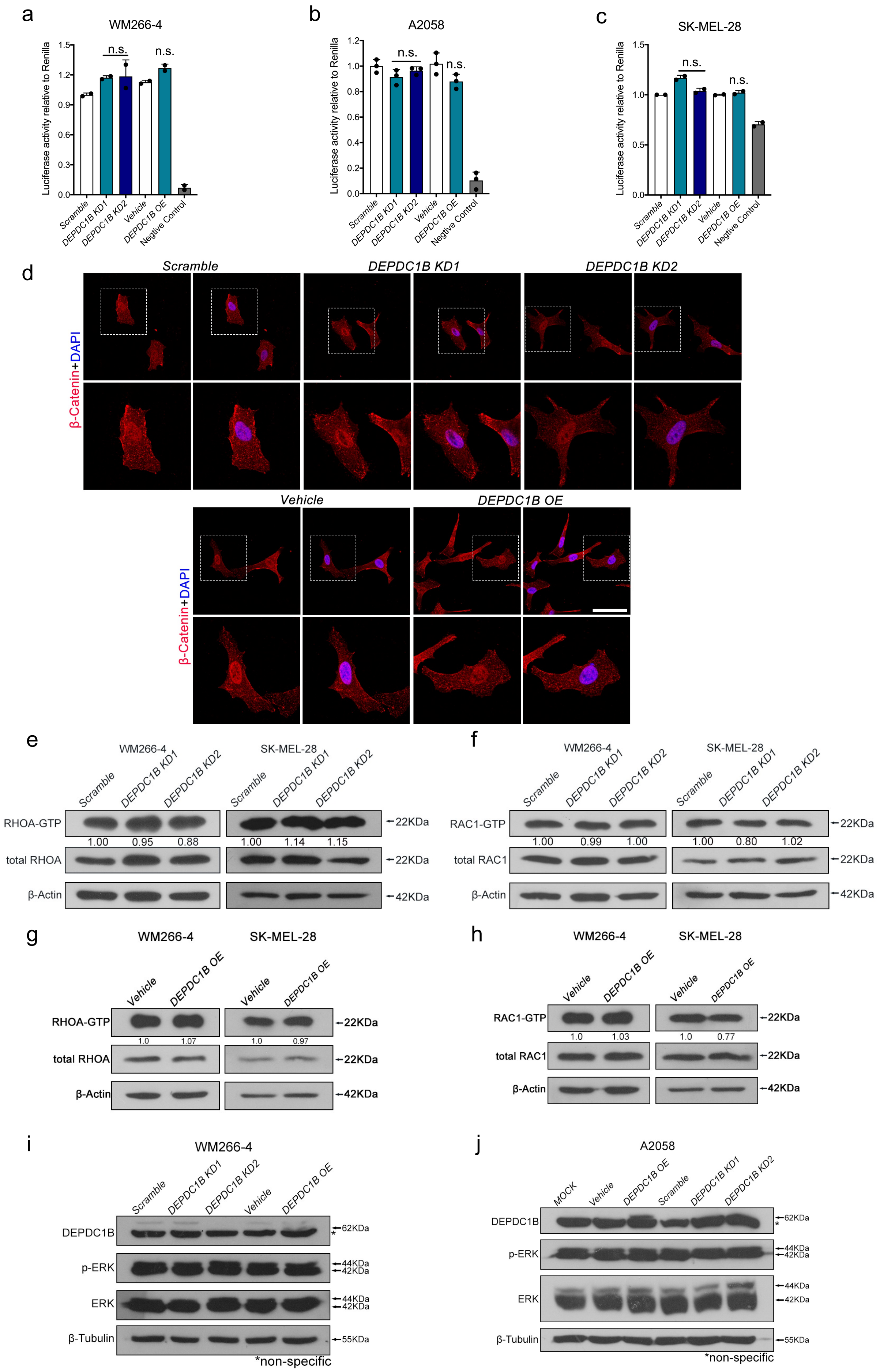
a
WM266-4
Luciferase activity relative to Renilla
n.s.
n.s.
Scramble
DEPDC1B KD1
DEPDC1B KD2
Vehicle
DEPDC1B OE
Negtive Control
b
A2058
n.s.
n.s.
c
SK-MEL-28
n.s.
n.s.
d
Scramble
DEPDC1B KD1
DEPDC1B KD2
β-Catenin+DAPI
Vehicle
DEPDC1B OE
e
WM266-4
SK-MEL-28
RHOA-GTP
1.00 0.95 0.88
1.00 1.14 1.15
total RHOA
β-Actin
22KDa
42KDa
f
RAC1-GTP
1.00 0.99 1.00
1.00 0.80 1.02
total RAC1
g
1.0 1.07
1.0 0.97
h
1.0 1.03
1.0 0.77
i
DEPDC1B
p-ERK
ERK
β-Tubulin
62KDa
44KDa
42KDa
55KDa
*non-specific
j
A2058
MOCK
*non-specific

Figure S4. DEPDC1B-mediated melanoma metastasis is independent of canonical Wnt signaling, Rho GTPase signaling and ERK activities. a-c) β-catenin TOPFlash luciferase reporter assays were carried out by transfection of M50 8x TOPFlash and Renilla constructs into melanoma cells. The negative control contained Renilla only. d) Representative immunofluorescence staining showed that β-catenin (red) in WM266-4 cells treated with the indicated constructs does not show altered subcellular localization. Cell nuclei were counter-stained with DAPI (blue). Scale bar = 50 μm. e&g) RHOA pull-down assays were conducted in melanoma cells treated with either *DEPDC1B KD* or *OE*. f&h) RAC1 pull-down assays were conducted in melanoma cells treated with either *DEPDC1B KD* or *OE*. i-j) WB results showed no marked alteration the phosphoration status of ERK in all the treatment groups. *Asterisk indicates non-specific band. Data are mean ± SD. n.s. when P>0.05 from ordinary one-way ANOVA.

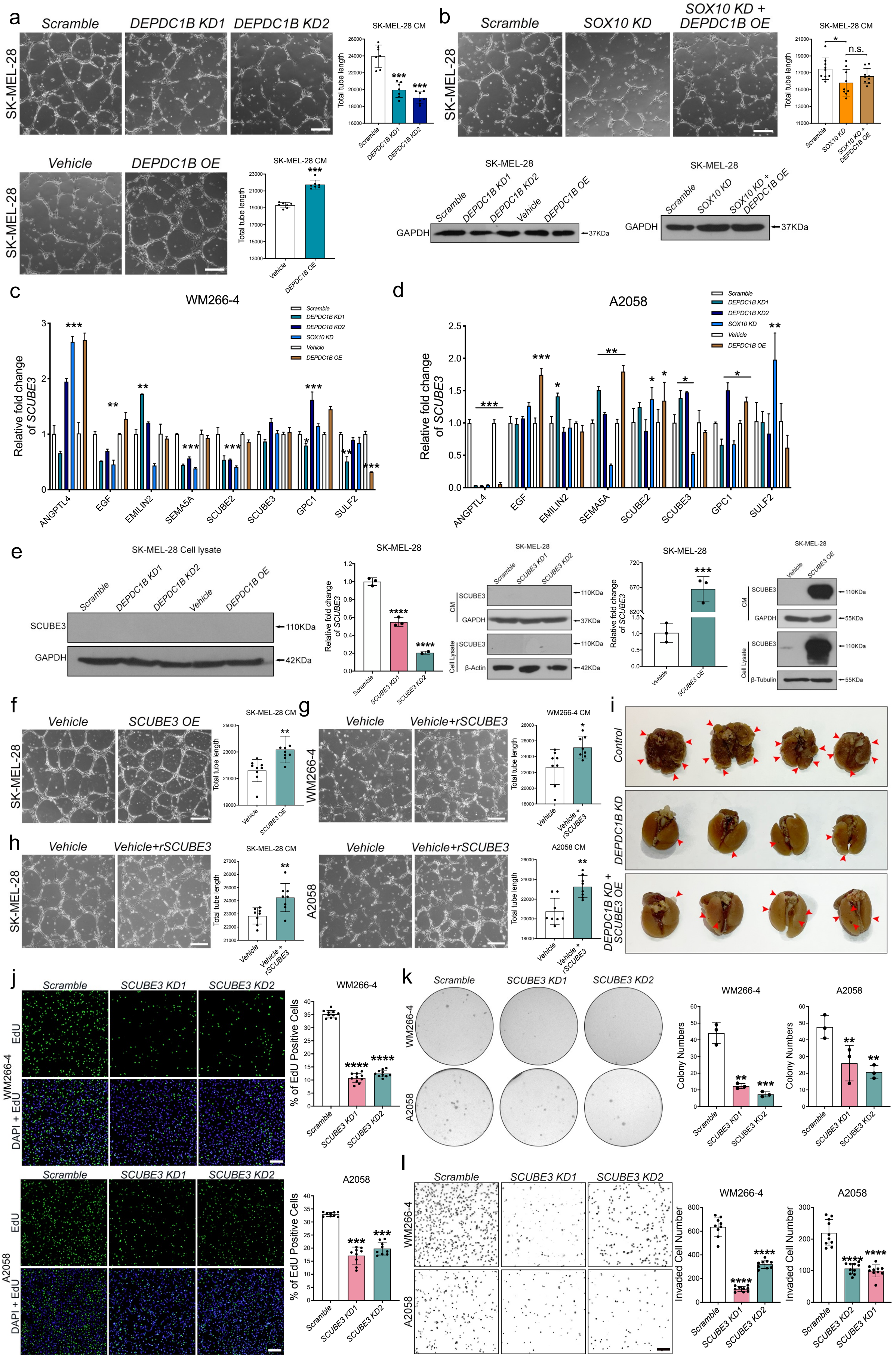
a
Scramble
DEPDC1B KD1
DEPDC1B KD2
SK-MEL-28
SK-MEL-28 CM
Total tube length
Vehicle
DEPDC1B OE
b
SOX10 KD
SOX10 KD + DEPDC1B OE
n.s.
GAPDH
37KDa
c
WM266-4
Relative fold change of SCUBE3
ANGPTL4
EGF
EMILIN2
SEMA5A
SCUBE2
SCUBE3
GPC1
SULF2
d
A2058
e
SK-MEL-28 Cell lysate
SCUBE3
110KDa
42KDa
SCUBE3 KD1
SCUBE3 KD2
SCUBE3 OE
CM
Cell Lysate
β-Actin
β-Tubulin
55KDa
f
g
h
i
Vehicle+rSCUBE3
WM266-4 CM
A2058 CM
Control
DEPDC1B KD
DEPDC1B KD + SCUBE3 OE
j
EdU
DAPI + EdU
% of EdU Positive Cells
k
Colony Numbers
l
Invaded Cell Number

Figure S5. DEPDC1B promotes melanoma angiogenesis through the secretion of SCUBE3. a&b) Representative images of HUVEC cells cultured with conditioned media from SK-Mel-28 cells treated with scramble, *DEPDC1B KD1*, *KD2*, vehicle control, DEPDC1B OE, SOX10 KD and DEPDC1B OE rescue. Western blot indicates equal seeding number. Scale bar = 200 µm. c-d) qPCR analysis of the potential candidate secreted factors. e) WB and qPCR analysis of SCUBE3 in SK-MEL-28 showing no endogenous SCUBE3 protein can be detected. f) In vitro angiogenesis assay using SK-MEL-28 parental cell CM and SCUBE3 OE CM. g&h) In vitro angiogenesis assay of treating HUVECs with recombinant protein SCUBE3 (rSCUBE3) in WM266-4, A2058 and SK-MEL-28 parental cell CM. Scale bar = 200 µm. i) Gross images of the mouse lungs from the indicated treatment groups. Red arrowheads indicate lung nodules. EdU staining (j), colony formation (k), and trans-well invasion assays (l) were performed in WM266-4 and A2058 cells treated with the indicated constructs. Scale bar = 200 µm. Data are mean ± SD. **P*<0.05; ***P*<0.01; ****P*<0.001; *****P*<0.0001 from unpaired Student′s t-test and ordinary one-way ANOVA.

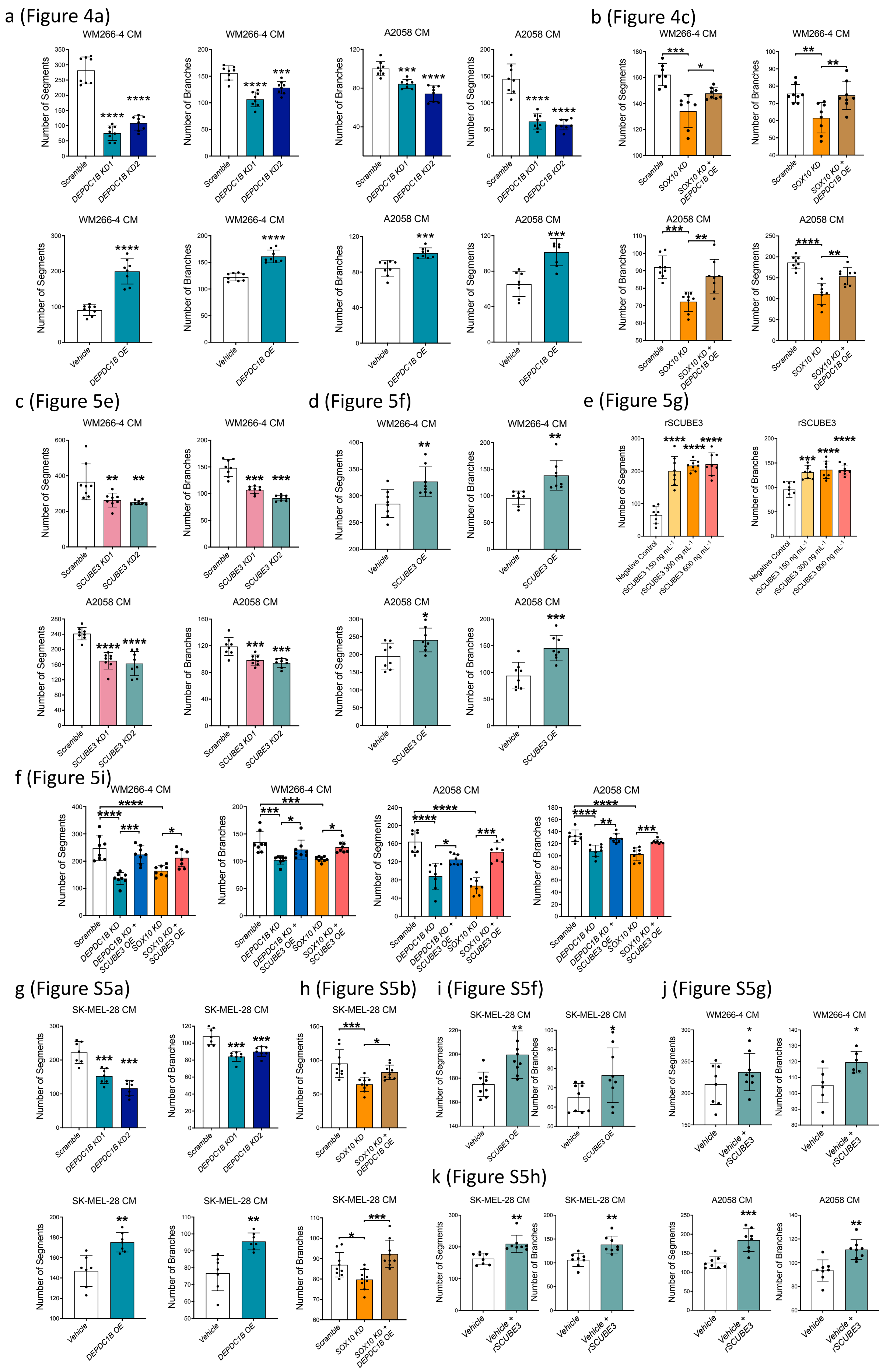
a (Figure 4a)
b (Figure 4c)
c (Figure 5e)
d (Figure 5f)
e (Figure 5g)
f (Figure 5i)
g (Figure S5a)
h (Figure S5b)
i (Figure S5f)
j (Figure S5g)
k (Figure S5h)
WM266-4 CM
A2058 CM
SK-MEL-28 CM
rSCUBE3
Number of Segments
Number of Branches
Scramble
DEPDC1B KD1
DEPDC1B KD2
DEPDC1B KD
DEPDC1B OE
Vehicle
SOX10 KD
SOX10 KD + DEPDC1B OE
SCUBE3 KD1
SCUBE3 KD2
SCUBE3 OE
DEPDC1B KD + SCUBE3 OE
SOX10 KD + SCUBE3 OE
Negative Control
rSCUBE3 150 ng mL⁻¹
rSCUBE3 300 ng mL⁻¹
rSCUBE3 600 ng mL⁻¹
Vehicle + rSCUBE3

Figure S6. HUVEC in vitro tube formation assay quantifications for number of segments and number of branches. a) Quantification results of the assays performed in Figure 4a. b) Quantification results of the assays performed in Figure 4c. c) Quantification results of the assays performed in Figure 5e. d) Quantification results of the assays performed in Figure 5f. e) Quantification results of the assays performed in Figure 5g. f) Quantification results of the assays performed in Figure 5i. g) Quantification results of the assays performed in Figure S5a. h) Quantification results of the assays performed in Figure S5b. i) Quantification results of the assays performed in Figure S5f. j) Quantification results of the assays performed in Figure S5g. k) Quantification results of the assays performed in Figure S5h. Data are mean ± SD. *P<0.05; **P<0.01; ***P<0.001; ****P<0.0001 from unpaired Student′s t-test and ordinary one-way ANOVA.

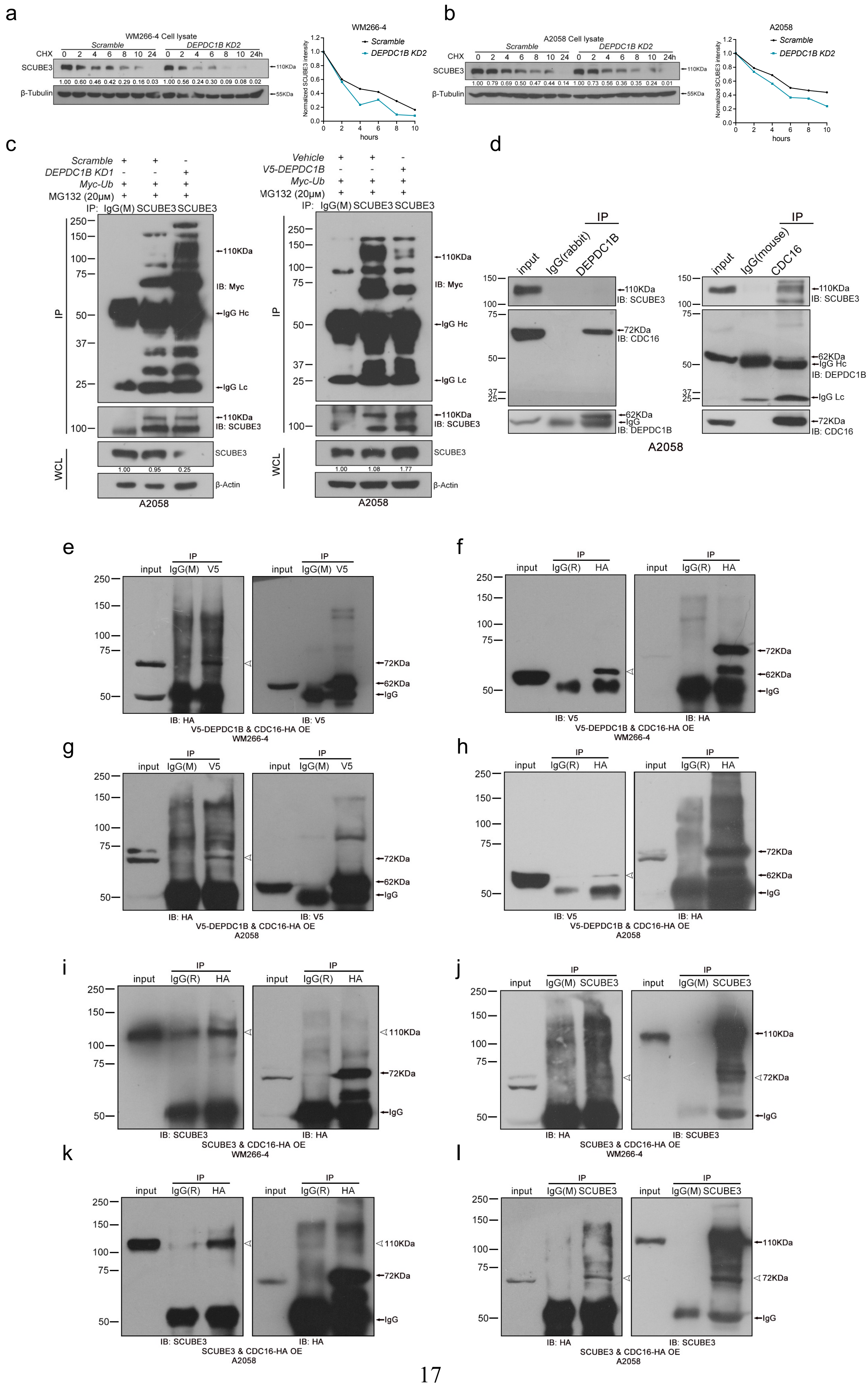
a
WM266-4 Cell lysate
Scramble
DEPDC1B KD2
CHX 0 2 4 6 8 10 24 0 2 4 6 8 10 24h
SCUBE3
110KDa
1.00 0.60 0.46 0.42 0.29 0.16 0.03 1.00 0.56 0.24 0.30 0.09 0.08 0.02
β-Tubulin
55KDa
WM266-4
Scramble
DEPDC1B KD2
Normalized SCUBE3 intensity
hours
b
A2058 Cell lysate
Scramble
DEPDC1B KD2
CHX 0 2 4 6 8 10 24 0 2 4 6 8 10 24h
SCUBE3
110KDa
1.00 0.79 0.69 0.50 0.47 0.44 0.14 1.00 0.73 0.56 0.36 0.35 0.24 0.01
β-Tubulin
55KDa
A2058
Scramble
DEPDC1B KD2
Normalized SCUBE3 intensity
hours
c
Scramble + + -
DEPDC1B KD1 - - +
Myc-Ub + + +
MG132 (20μm) + + +
IP: IgG(M) SCUBE3 SCUBE3
110KDa
IB: Myc
IgG Hc
IgG Lc
110KDa
IB: SCUBE3
IP
WCL
SCUBE3
1.00 0.95 0.25
β-Actin
A2058
Vehicle + + -
V5-DEPDC1B - - +
Myc-Ub + + +
MG132 (20μm) + + +
IP: IgG(M) SCUBE3 SCUBE3
110KDa
IB: Myc
IgG Hc
IgG Lc
110KDa
IB: SCUBE3
SCUBE3
1.00 1.08 1.77
β-Actin
A2058
d
IP
input IgG(rabbit) DEPDC1B
110KDa
IB: SCUBE3
72KDa
IB: CDC16
62KDa
IgG
IB: DEPDC1B
A2058
IP
input IgG(mouse) CDC16
110KDa
IB: SCUBE3
62KDa
IgG Hc
IB: DEPDC1B
IgG Lc
72KDa
IB: CDC16
e
input IgG(M) V5
IP
IB: HA
IB: V5
72KDa
62KDa
IgG
V5-DEPDC1B & CDC16-HA OE
WM266-4
f
input IgG(R) HA
IP
IB: V5
IB: HA
72KDa
62KDa
IgG
V5-DEPDC1B & CDC16-HA OE
WM266-4
g
input IgG(M) V5
IP
IB: HA
IB: V5
72KDa
62KDa
IgG
V5-DEPDC1B & CDC16-HA OE
A2058
h
input IgG(R) HA
IP
IB: V5
IB: HA
72KDa
62KDa
IgG
V5-DEPDC1B & CDC16-HA OE
A2058
i
input IgG(R) HA
IP
IB: SCUBE3
IB: HA
110KDa
72KDa
IgG
SCUBE3 & CDC16-HA OE
WM266-4
j
input IgG(M) SCUBE3
IP
IB: HA
IB: SCUBE3
110KDa
72KDa
IgG
SCUBE3 & CDC16-HA OE
WM266-4
k
input IgG(R) HA
IP
IB: SCUBE3
IB: HA
110KDa
72KDa
IgG
SCUBE3 & CDC16-HA OE
A2058
l
input IgG(M) SCUBE3
IP
IB: HA
IB: SCUBE3
110KDa
72KDa
IgG
SCUBE3 & CDC16-HA OE
A2058

Figure S7. DEPDC1B stabilizes SCUBE3 through inhibition of ubiquitin-mediated degradation pathway. a&b) Cycloheximide chase assay was conducted in WM266-4 and A2058 cells treated with *DEPDC1B KD2*. c) A2058 cells were treated with *DEPDC1B KD1* and *Myc-Ubiquitin OE* for 72 hours and MG132 (20 μM) for 6 hours prior to SCUBE3 immunoprecipitation. The amount of cell lysate used for IP was normalized proportionally by SCUBE3 whole cell protein lysate. Anti-Myc was used to detect the ubiquitylated level of SCUBE3. d) Endogenous co-immunoprecipitation in A2058 cells showing the interaction between DEPDC1B and CDC16, CDC16 and SCUBE3 but not DEPDC1B and SCUBE3. e-h) V5-DEPDC1B and CDC16-HA were over-expressed in WM266-4 and A2058 cells. White arrowheads indicate the position of the specific IP bands. Co-immunoprecipitation was carried out by V5 antibody pull-down (e&g) and HA antibody pull-down (f&h) separately. i-l) SCUBE3 and CDC16-HA were over-expressed in WM266-4 and A2058 cells. Arrowheads indicate the position of the specific IP bands. Co-immunoprecipitation was done by HA antibody pull-down (i&k) and SCUBE3 antibody pull-down (j&l) separately.

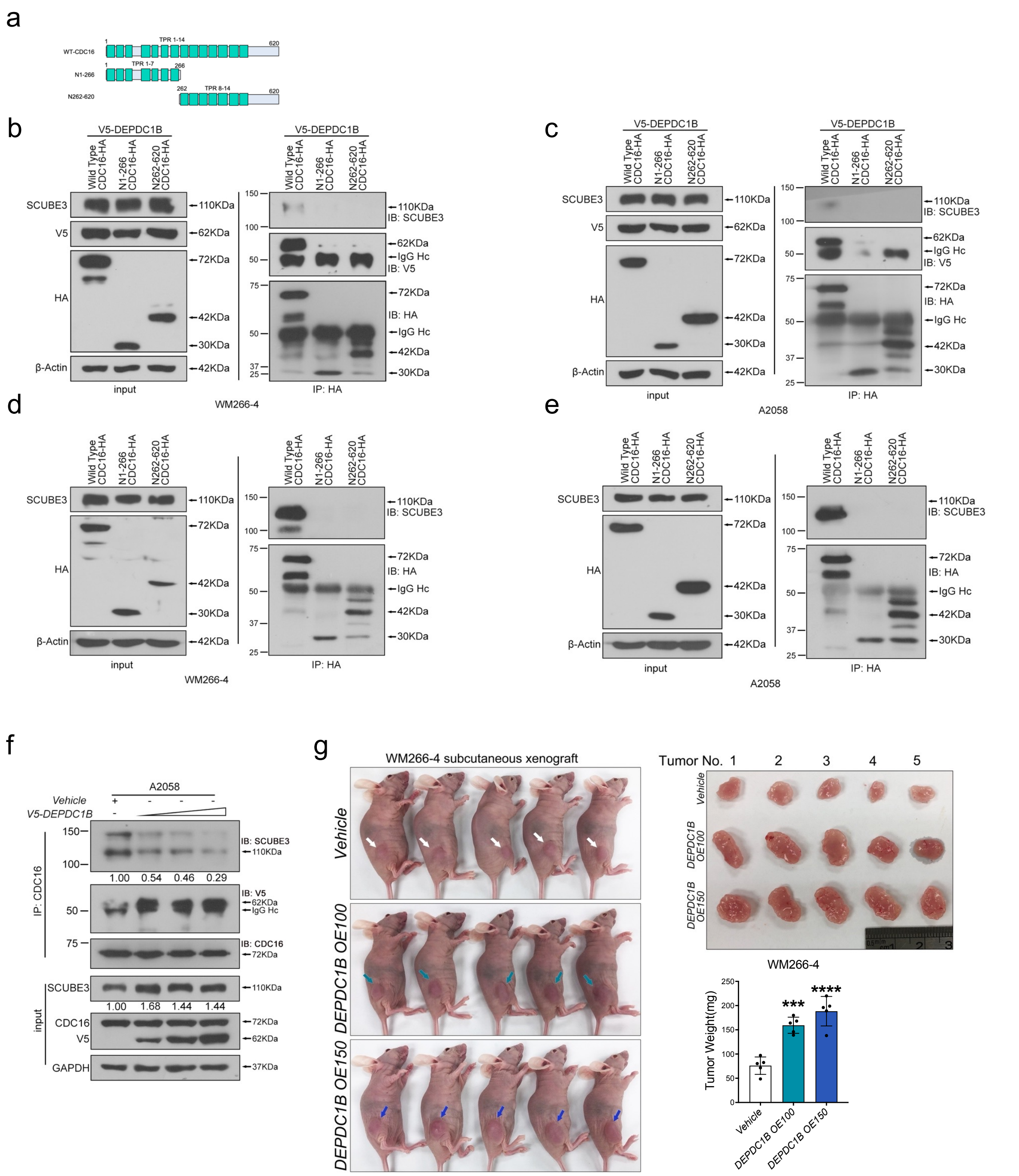

Figure S8. DEPDC1B interacts with full-length CDC16. a) Schematic showing the CDC16 full-length and two truncated constructs carrying TPR 1-7 domain and TPR 8-14 domain. The three constructs were expressed in WM266-4 and A2058 cells with (b&c) or without (d&e) V5-DEPDC1B overexpression. Co-immunoprecipitations were performed using HA antibody for pull down. f) Competitive co-immunoprecipitation among DEPDC1B, CDC16 and SCUBE3 performed in A2058 cell line. g) Gross image of the mice and dissected subcutaneous xenograft

tumors. The weight of xenograft tumors was measured and analyzed. Data are mean ± SD. *P<0.05; **P<0.01; ***P<0.001; ****P<0.0001 from ordinary one-way ANOVA.

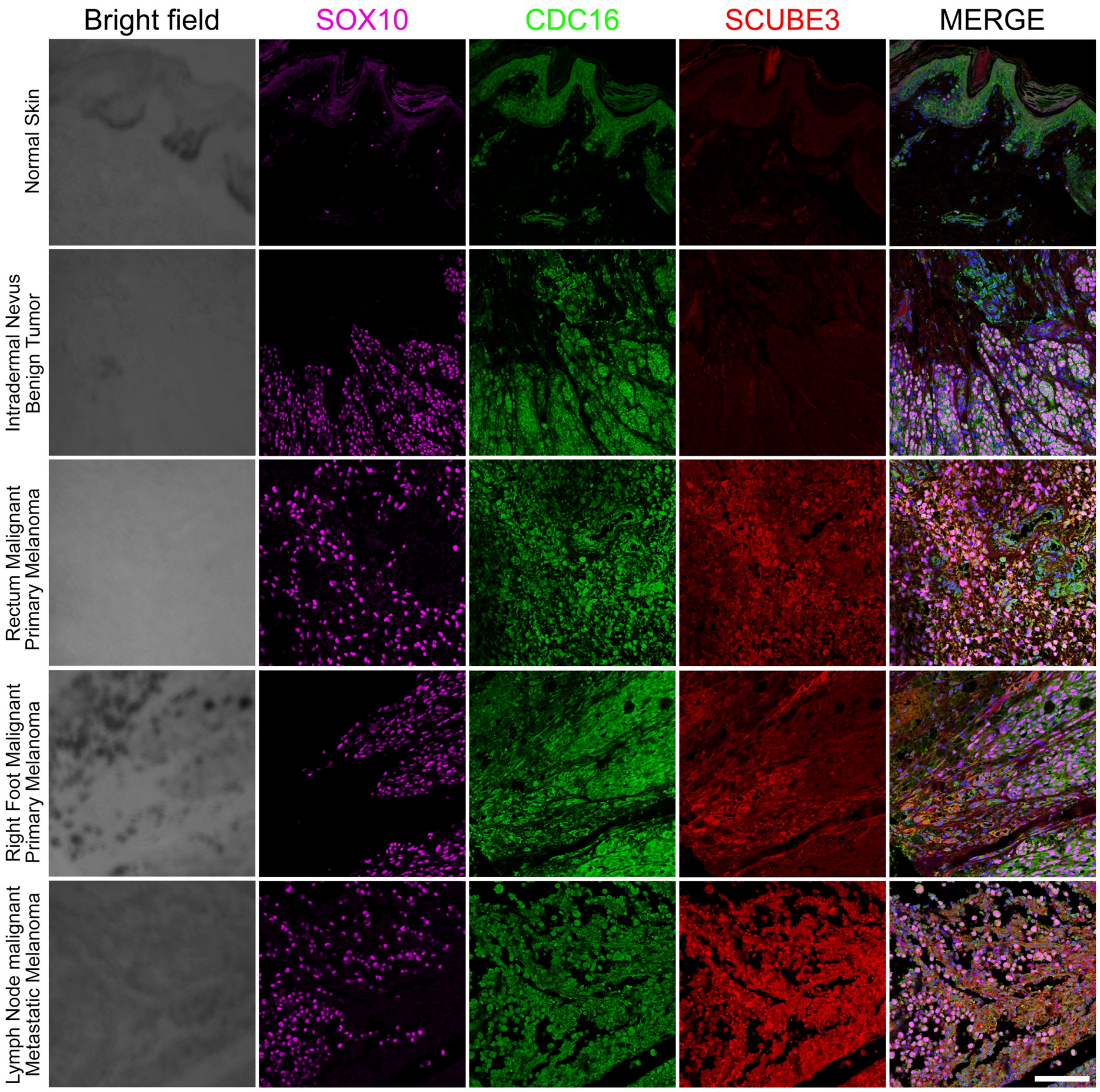

Figure S9. TMA immunofluorescence staining using anti-SCUBE3 (red), anti-CDC16 (green) and anti-SOX10 (magenta) and DAPI counterstaining for nuclei (blue in merge). Scale bar = 50 μm.

Supporting Tables

Table S1-4. Results of Mass Spectrometry (MS).

Table S1: MS analysis of conditioned media collected from WM266-4 cells treated with *Scramble*, *DEPDC1B KD1* and *DEPDC1B KD2*.

Table S2: MS analysis of conditioned media collected from WM266-4 cells treated with *Vehicle*, *DEPDC1B OE*.

Table S3: MS analysis of lysates immunoprecipitated by IgG, DEPDC1B and V5 antibodies in WM266-4.

Table S4: MS analysis of lysates immunoprecipitated by IgG, DEPDC1B and V5 antibodies in A2058.

(Attached as separate excel files)

Table S5. Cell lines and culture methods

Cell lines	Stage	Genetic feature	Trypsin concentration	Passage Ratio	Culture medium
WM266-4	Metastasis	$BRAF^{V600D}$	0.125%	1:5	EMEM+10%FBS
A2058	Metastasis	$BRAF^{V600E}$	0.25%	1:5	EMEM+10%FBS
SK-MEL-28	Primary	$BRAF^{V600E}$	0.25%	1:8	DMEM+10%FBS
HUVEC	-	-	0.125%	1:8	DMEM+10%FBS
293T	-	-	0.025%	1:3	M200+LSGS

Table S6. PCR primers used in the study

Primers	Sequence (5' to 3')
F' BamH1-V5-DEPDC1B	CGCGGATCCGCGATGGGTAAGCCTATCCCTAACCCTCTCCT CGGTCTCGATTCTACGATGGAG CATCGCATCGTG
F' BamH1-V5-DEPDC1B-N107	CGCGGATCCGCGATGGGTAAGCCTATCCCTAACCCTCTCCT CGGTCTCGATTCTACGCCTCCTTCTTCACCCCTGAAA
F' BamH1-V5-DEPDC1B-N177	CGCGGATCCGCGATGGGTAAGCCTATCCCTAACCCTCTCCT CGGTCTCGATTCTACGCTGACAGAGGCCAATGTA
F' BamH1-V5-DEPDC1B-N401	CGCGGATCCGCGATGGGTAAGCCTATCCCTAACCCTCTCCT CGGTCTCGATTCTACGACCTCTATAGAGGAGCGT
R' Xba1-DEPDC1B	GCTCTAGAGCTTACATTCGAAAACTTCT
R' Xba1-DEPDC1B-N151	GCTCTAGATTAATTCATCACAACTGGCCT
F' BamH1-HA-SOX10	CGCGGATCCGCGATGTACCCATACGATGTTCCAGATTACG CTATGGCGGAGGAGCAGGAC
R' Xba1-SOX10	GCTCTAGAGCTTAGGGCCGGGACAGTGT
F' EcoR1-SCUBE3	CGGAATTCATGGGCTCGGGGCGCGTAC
R' Xba1-SCUBE3	GCTCTAGACTATTTGTAGGGCCTCAG
F' EcoR1-CDC16	CGGAATTCATGAACCTAGAGCGGCTGC
F' EcoR1-CDC16-N262-620	CGGAATTCATGCCTTTCCATGCAAGTTGTT
R' BamH1-HA-CDC16-N266	CGGGATCCTCAAGCGTAATCTGGAACATCGTATGGGTAAC TTGCATGGAAAGGATC

Table S7. List of shRNA oligo target sequences

Gene	Sequence (5' to 3')
Scramble	CCTAAGGTTAAGTCGCCCTCG
DEPDC1B KD1	GTACUGGGTTTGTTACAGA
DEPDC1B KD2	GATCATATGGCTCTCAGGATT
SOX10	GACTTCGGCAACGTGGACATT
SCUBE3 KD1	GCAGAGCTGTGTCAACATGAT
SCUBE3 KD2	CCATCCTCCATTACCACTTAT
CDC16 KD1	ACGCTTGTAGAGCTGAATAAA
CDC16 KD2	CTATCTCATGGTCGGTCATAA

Table S8. List of qPCR primers sequences

Gene	Direction	primers (5' to 3')
DEPDC1B	Forward	TTTGGATATGGTTTCAGGGG
	Reverse	ACATCAAGGGAAAATGGGGT
SOX10	Forward	GACCAGTACCCGCACCTG
	Reverse	CGCTTGTCACTTTCGTTCAG
SCUBE3	Forward	GCTGTGTCAACATGATGGGC
	Reverse	CCGCTGGATACAGGTATGCTG
CDC16	Forward	ATGCTGAGGCCTTGGATTACC
	Reverse	TCTCGCCTAAGACCAAGGGC
36B4	Forward	GTGATGTGCAGCTGATCAAGACT
	Reverse	GAAGACCAGCCCAAAGGAGA
SCUBE2	Forward	CAGGCAGAGTCCTGTGGAGT
	Reverse	TAAAATGCAGCGTTCTCGTG
SULF2	Forward	GACCCCTACCAGCTGATGAA
	Reverse	GCTTGTAACCCTTGCAGCTC
ANGPTL4	Forward	AAAGAGGCTGCCCGAGAT
	Reverse	TCTCCCCAACCTGGAACA
EGF	Forward	GTGCAGCTTCAGGACCACAA
	Reverse	CATGTGTCGAATATCTTGAG
EMILIN2	Forward	GGGCGTTGTCCTCTTTA
	Reverse	CGTAGGCGTCTCTCTCG
GPC1	Forward	TGCCCTGACTATTGCCGAA
	Reverse	CATGGAGTCCAGGAGGTTCCT
SEMA5A	Forward	GATCTATGGCATCTTTACCACCA
	Reverse	TGGCGCTCAGGTTGAAGAC

Table S9. List of antibodies used and the dilution scale

Primary Antibody	Host	IB dilution	IF dilution	Company	Catalog Number
DEPDC1B	Rabbit	1:1000	1:1000	GenScript	Customed
SOX10	Goat	1:500	1:50	ThermoFisher	PA5-40697
V5	Mouse	1:5000	1:1000	Invitrogen	R960-25
SCUBE3	Mouse	1:500	1:100	Santa Cruz	sc-514696
CDC16	Mouse	1:500	1:100	Santa Cruz	sc-365636
CDC16	Rabbit	1:1000	1:50	ABClonal	A7197
CD31	Goat	-	1:100	Santa Cruz	sc-1506
GAPDH	Rabbit	1:5000	-	Santa Cruz	sc-25778
β-Actin	Mouse	1:2000	-	Sigma	A2228
β-Tublin	Rabbit	1:10000	-	CST	2146

Table S10. ChIP qPCR primers

primers	Forward primer (5' to 3')	Reverse primer (5' to 3')
motif 1	CATACTTTGGGGCCGTGTC	ATCTTCTAGTGACTCCCTAG
motif 2	GTGAAGTCTGAAGAAGTGGC	GCTGCCTGACATTCCTGATG
motif 3&4	ACAACCCTATTGCCGAGGAC	ATAAAGAGCGGGAAGGGAGC
motif 5	GTGGAAGATAGGGTTACTTCG	CAGCAGGCCACAAAGATTTCT
MIA	TGGGCTGTTTCTGGTAATCA	CACCTTGGAATTTCCTGTGC
GAPDH	TACTAGCGGTTTTACGGGCG	TCGAACAGGAGGAGCAGAGAGCGA
